# Type II tRNA cleavage by SLFN14 endoribonuclease variants linked to inherited thrombocytopenia drives global translational repression

**DOI:** 10.1371/journal.pbio.3003830

**Published:** 2026-05-29

**Authors:** Chengchao Ding, Xinyi Ashley Liu, Fushun Zhang, Saori Uematsu, Shu-Bing Qian, Yan Xiang

**Affiliations:** 1 Department of Microbiology, Immunology‌‌ and Molecular Genetics, Joe R. & Teresa Lozano Long School of Medicine, The University of Texas at San Antonio, San Antonio, Texas, New York, United States of America; 2 Department of Infectious Disease, The First Affiliated Hospital of USTC, Division of Life Sciences and Medicine, University of Science and Technology of China, Hefei, Anhui, China; 3 Division of Nutritional Sciences, Cornell University, Texas, New York, United States of America; Yale University, Connecticut, UNITED STATES OF AMERICA

## Abstract

Schlafens proteins (SLFNs) are interferon-inducible regulators of RNA metabolism that influence antiviral defense and cell fate. Human SLFN14 is a ribosome-associated endoribonuclease whose pathogenic variants cause autosomal dominant inherited thrombocytopenia (IT), but the molecular basis of this disorder remains unclear. Here, using HEK293T cells expressing human SLFN14 variants, we show that SLFN14 represses global protein synthesis through selective cleavage of type II tRNAs. IT-linked mutations alter SLFN14 RNA substrate specificity, enhancing depletion of type II tRNAs while reducing rRNA cleavage. This shift promotes ribosome stalling at codons decoded by type II tRNAs, triggering global translational arrest, stress signaling, and cell death. These findings reveal how altered RNA targeting by SLFN14 can drive disease and highlight selective tRNA targeting as a mechanism than regulates translation and cell fate.

## Abstract 2

Pathogenic variants in the ribosome-associated endoribonuclease SLFN14 cause inherited thrombocytopenia, but the molecular basis of this disorder is unclear. This study shows that type II tRNA cleavage by mutant SLFN14 drives global translational repression and cell death.

## Introduction

Schlafens‌‌ (SLFNs) are a family of vertebrate genes with important roles in immune regulation, antiviral defense, and cellular sensitivity to DNA-targeted anticancer agents [1,2]. Humans encode five SLFN proteins, SLFN5, SLFN11, SLFN12, SLFN13, and SLFN14. Among these, SLFN14 is unique for its association with inherited thrombocytopenia (IT). SLFN14 is predominantly expressed in hematopoietic lineages, especially megakaryocyte (MK) and erythroid precursors [3,4]. Heterozygous missense mutations in SLFN14 cause an autosomal dominant form of IT, characterized by defective platelet function and excessive bleeding [5–8]. In addition to its role in hematopoiesis, SLFN14 has been identified as an interferon-stimulated antiviral factor that inhibits replication of influenza A virus [9], varicella-zoster virus, and HIV-1 [10–12].

SLFNs are multidomain proteins that can be classified into subgroups based on domain architecture [1]. Group III SLFNs, including SLFN5, SLFN11, SLFN13, and SLFN14, contain an N-terminal RNase domain, a central SWADL or linker region, and a C-terminal helicase-like domain, whereas group II SLFNs such as SLFN12 lack the helicase-like domain. Structures of several full-length SLFNs and isolated RNase domains have been determined [11–17], providing important insights into SLFN enzymatic activity and RNA substrate recognition. These studies revealed that the RNase domain adopts a U-shaped pseudodimeric architecture composed of N- and C-lobes, which together form a positively charged central valley capable of engaging RNA substrates. In vitro, RNase domains from multiple SLFNs display relatively broad and variable RNA substrate specificity. In particular, purified SLFN14 RNase domain cleaves rRNA, tRNA, and diverse structured RNAs containing double-stranded regions [11–13].

Notably, most IT-associated SLFN14 mutations, including K218E, K219N/E, V220D, and R223W, cluster within the RNA-binding cleft of the RNase domain, although a more recent study identified an IT-associated frameshift variant, T853fs, in the C-terminal helicase domain [18]. Most IT-associated mutations in the RNase domain reduce RNA cleavage activity in vitro [11,12]. For example, the K219N mutation decreases cleavage efficiency against both rRNA and tRNA [12]. However, despite extensive biochemical and structural characterization, the physiological RNA substrates that mediate SLFN14 cellular function remain unclear, and the mechanisms by which IT-linked SLFN14 variants drive disease pathogenesis have not been resolved.

In this study, we show that SLFN14 represses global protein synthesis and identify selective cleavage of type II tRNAs as the primary mechanism underlying this activity. We further demonstrate that IT-linked mutations reprogram SLFN14 RNA substrate specificity, enhancing depletion of type II tRNAs while reducing rRNA cleavage. This altered RNA targeting promotes ribosome stalling, translational arrest, and cellular stress, providing mechanistic insight into the pathogenesis of SLFN14-associated thrombocytopenia.

## Results

### SLFN14 inhibits global protein synthesis, an effect exacerbated by IT-associated mutations

**Fig 1:**
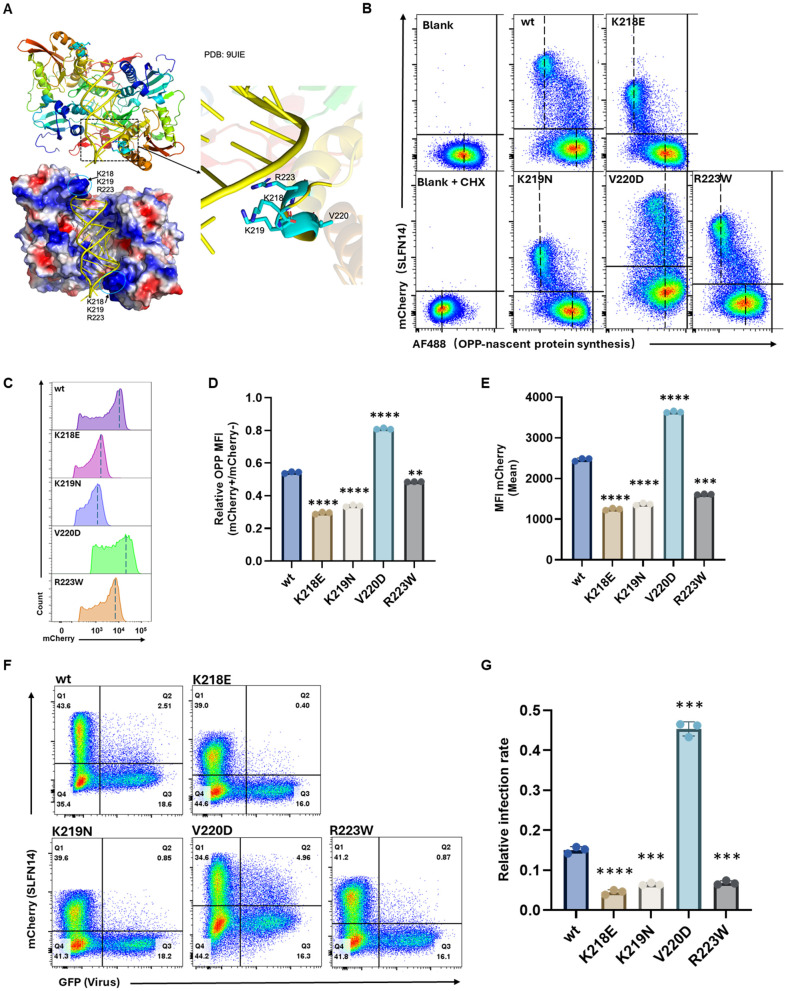
SLFN14 inhibits global protein synthesis and viral replication, effects exacerbated by IT-associated mutations. **A)** Location of IT-associated mutations in the structure of the human SLFN14 RNase domain bound to dsRNA (PDB: 9UIE). The RNase domain is shown in both cartoon and electrostatic surface representations, highlighting the RNA-binding cleft. A close-up view of IT-associated residues relative to the bound RNA is also shown. **(B–E)** HEK293T cells were transfected with mCherry-P2A-SLFN14 variants. At 16 h post-transfection, nascent protein synthesis was measured by OPP incorporation for 30 min. Untransfected cells and cells treated with CHX were included as controls. **(B)** Representative flow cytometry plots showing OPP levels relative to mCherry expression. The vertical dashed line indicates the mean fluorescence intensity (MFI) of the cell populations. **(C)** Histogram of mCherry-P2A–SLFN14 expression. **(D)** Quantification of nascent protein synthesis in SLFN14-expressing (mCherry^+^) cells relative to non-transfected (mCherry^−^) cells. **(E)** Quantification of mCherry MFI. **(F-G)** HEK293T cells were transfected with mCherry-P2A-SLFN14 variants for 36 h and infected with VACV/GFP^+^ for 15 **h. (F)** Representative flow cytometry plots showing GFP expression in SLFN14-expressing vs. untransfected cells. Percentages of cells in each quadrant are indicated. **(G)** Relative infection (GFP^+^) rates between SLFN14-expressing (mCherry^+^) and nontransfected (mCherry^−^) cell populations were quantified from flow cytometry data. Rate = [*Q*2/(*Q*2 + *Q*1)]/[*Q*3/(*Q*3 + *Q*4)]. The data underlying this Figure can be found in S1 Data.

A consistent observation in SLFN14-linked IT is the dramatic reduction of SLFN14 expression in patient-derived platelets and in transfected cells [5–7], which has been attributed to a dominant-negative effect of mutants on wild-type (WT) protein synthesis or stability. To examine the broader impact of SLFN14 on translation, we transiently expressed SLFN14 in HEK293T cells and measured nascent protein synthesis using O-propargyl-puromycin (OPP) labeling. An mCherry tag was placed at the N-terminus of SLFN14 followed by a P2A ribosomal skipping sequence, allowing single-cell monitoring of SLFN14 expression by flow cytometry. Cells with no or low expression of WT SLFN14 showed protein synthesis comparable to untransfected controls (Fig 1B). By contrast, cells with high levels of WT SLFN14 exhibited complete suppression of protein synthesis, similar to cycloheximide (CHX)-treated cells (Fig 1B), indicating that SLFN14 overexpression potently inhibits translation.

Strikingly, even cells expressing low levels of IT-linked variants K218E or K219N displayed little or no protein synthesis, demonstrating enhanced translational inhibition (Fig 1B). The R223W variant also enhanced the inhibition, whereas the V220D variant reduced the inhibition. The extent of inhibition by the SLFN14 variants followed the order K218E ≈ K219N > R223W > WT > V220D (Fig 1D). Notably, V220D has been reported to differ from other IT-linked mutations in that its expression did not reduce WT SLFN14 levels in transfected cells [5]. Consistent with this distinction, structural analysis shows that V220 is oriented away from the RNA-binding interface [11], in contrast to K218, K219, and R223 (Fig 1A). The effect of IT-linked mutations on protein synthesis was also reflected by mCherry expression levels in cells (Fig 1C and 1E), as mCherry mean fluorescence intensity (MFI) correlated with OPP MFI. Inhibition of protein synthesis required SLFN14 RNase activity, as the catalytic site mutation E206A abolished the ability of both WT and K219N to suppress translation (S1B Fig). SLFN14 with an N-terminal FLAG tag (FLAG-SLFN14) also inhibited global protein synthesis (S1D Fig), indicating that its function is not affected by N-terminal tagging.

To precisely control SLFN14 expression, we generated doxycycline (Dox)-inducible 293T cell lines expressing either WT or K219N mCherry-SLFN14. After 12 hours (h) of induction, protein synthesis was strongly reduced but not completely abolished, with the K219N variant showing greater inhibition than WT (S2A and S2B Fig). After 24 h, both WT and K219N blocked protein synthesis, comparable to CHX treatment. Western blotting showed that the SLFN14^K219N^ protein level was substantially lower than WT (S2F Fig), reflecting its stronger inhibition of cellular protein synthesis.

### SLFN14 inhibits vaccinia virus replication, with enhanced effects by IT-associated mutations

To test the effects of IT-associated mutations on antiviral activity, we examined their ability to restrict vaccinia virus (VACV), a cytoplasmic DNA virus. HEK293T cells were transfected with mCherry-P2A-SLFN14 constructs and infected with a GFP-expressing VACV. WT SLFN14 inhibited VACV replication, as indicated by reduced GFP expression in mCherry^+^ cells (Fig 1F). The K218E, K219N, and R223W variants displayed even stronger antiviral activities, whereas V220D showed reduced antiviral activity compared to WT. The relative antiviral activities followed the order K218E ≈ K219N ≈ R223W > WT > V220D (Fig 1G), which parallels their effects on global protein synthesis. Consistently, the catalytic site mutation E206A abolished the ability of both WT and K219N SLFN14 to inhibit viral replication (S1C Fig).

We further examined antiviral activity against VACV with the Dox-inducible SLFN14 cell lines. Upon Dox induction, both WT and K219N lines exhibited a significant decrease in infection rates (%GFP+ cells) compared to uninduced controls (S2C and S2D Fig). Viral titers measured by plaque assay confirmed that viral growth was completely abolished, with K219N cells showing a slightly greater reduction in viral titers than the WT cells at 24 h post infection (S2E Fig).

### IT-associated mutations enhance SLFN14-mediated depletion of type II tRNAs while reducing rRNA cleavage

**Fig 2:**
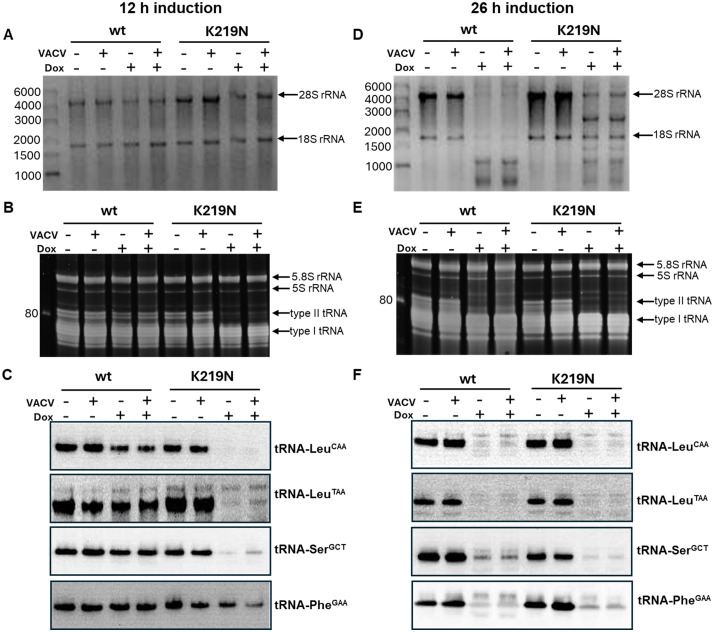
IT-linked mutations enhance SLFN14-mediated depletion of type II tRNAs while reducing rRNA cleavage. Analysis of total RNA from Dox-inducible 293T cells expressing WT or K219N SLFN14 after 12 h **(A–C)** or 26 h **(D–F)** of induction. **(A,D)** RNA was resolved on 2% agarose gel and stained with SYBR Safe DNA Gel Stain. RNA molecular weight markers (in nt) are shown. **(B,E)** RNA was resolved on a polyacrylamide/urea gel and stained with SYBR Gold stain. **(C,F)** Northern blot analysis using probes specific for the indicated tRNAs. VACV indicates infection with vK1^−^C7^−^/GFP^+^ VACV. The raw images underlying this Figure can be found in S1 Raw Images.

To investigate how IT-linked mutations alter SLFN14-mediated RNA processing, we analyzed total RNA from Dox-inducible WT and K219N cells. After induction for 12 hours, rRNA degradation was not detectable in either WT or K219N cells by nucleic acid staining (Fig 2A). Surprisingly, type II tRNAs, with the longer variable loop, were found to be completely depleted in K219N cells while only slightly reduced in WT cells (Fig 2B). This was confirmed by Northern blotting against the specific type II tRNAs (tRNA-Leu^CAA^, tRNA-Leu^TAA^, and tRNA-Ser^GCT^). The northern blot also revealed that tRNA-Phe, a type I tRNA, was modestly reduced in K219N cells but remained unchanged in WT cells (Fig 2C).

After induction of SLFN14 for 26 hours, rRNA degradation was prominent in WT cells but much weaker in K219N cells (Fig 2D). Type II tRNAs were also greatly reduced in WT cells, although their levels remained higher than in K219N cells (Fig 2E). While SYBR Gold staining could not clearly resolve changes in overall level of type I tRNAs, northern blot demonstrated that tRNA-Phe was strongly reduced at this time point (Fig 2F). In these experiments, an additional set of cells were also infected with VACV, and the results showed that the viral infection did not alter the RNA degradation by SLFN14.

Together, these results indicate that while SLFN14 targets both rRNA and tRNAs in cells, the K219N variant shifts substrate preference toward type II tRNAs.

### SLFN14 causes ribosomal stalling at codons decoded by type II tRNAs, an effect exacerbated by IT-linked mutations

**Fig 3:**
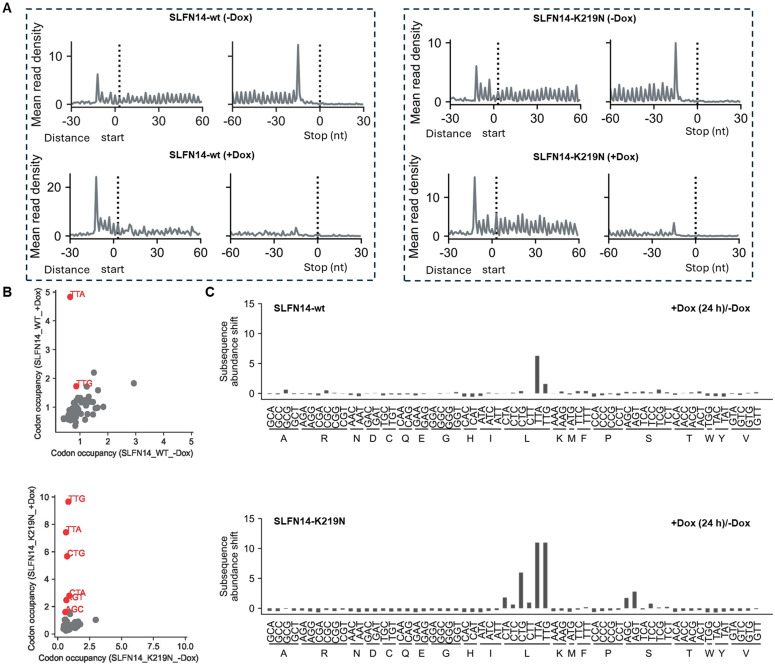
SLFN14 causes ribosomal stalling at codons decoded by type II tRNAs, an effect exacerbated by IT-linked mutations. Dox-inducible 293T cell lines expressing WT or K219N SLFN14 were left untreated or induced with Dox for 24 h before performing Ribo-seq. Global codon occupancy across all cellular transcripts was analyzed. **(A)** Aggregation plots around start and stop codons. All the samples showed clear 3-nt periodicity. **(B)** Codon occupancy analysis in Dox-induced cells relative to uninduced cells for each codon. Codons marked in red indicate those with large subsequence abundance shifts (>1) under Dox treatment. (**C)** Subsequence abundance shifts of individual codons in Dox-induced cells relative to uninduced cells. The data underlying this Figure can be found in S1 Data.

To assess the impact of SLFN14 on translation at codon resolution, we performed ribosome profiling (ribo-seq). Induction of WT SLFN14 for 24 hours caused ribosome stalling only at leucine codons TTA and TTG. In comparison, the K219N variant caused a stronger stalling at these codons, as well as stalling at leucine codons CTA and CTG and serine codons AGC and AGT (Fig 3). Both serine codons are decoded by tRNA-Ser^GCT^. The pattern and magnitude of ribosome stalling correlated with the extent of tRNA depletion in WT and K219N cells (Fig 2), confirming that SLFN14 promotes ribosome stalling through preferential depletion of type II tRNAs.

### IT-associated mutation causes increased cytotoxicity and eIF4E phosphorylation

**Fig 4:**
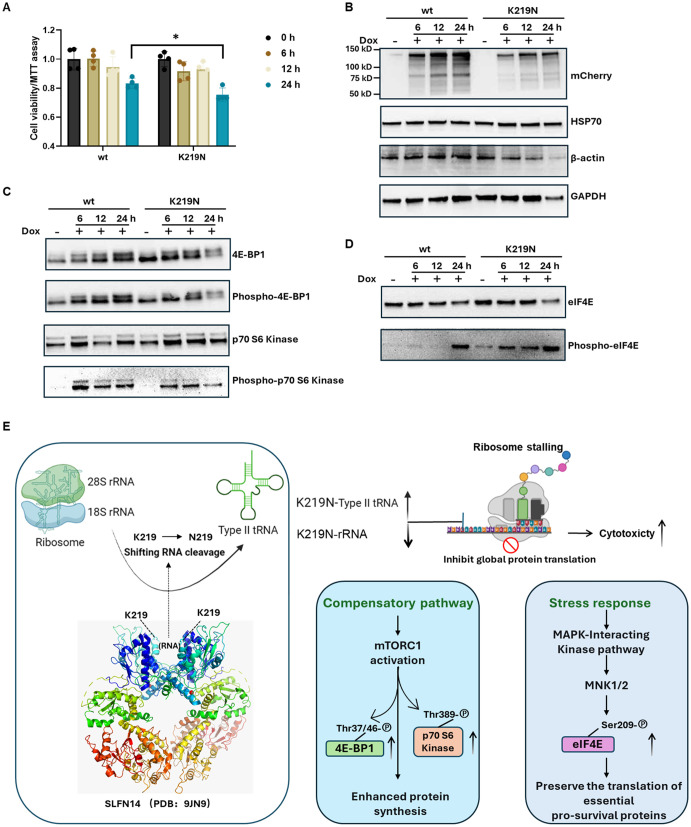
The K219N mutation in SLFN14 causes increased cytotoxicity and eIF4E phosphorylation. **(A)** 293T cells were induced to express SLFN14 for 6, 12, or 24 h, and cell viability was assessed using the MTT assay. **(B)** Western blot analysis of mCherry-SLFN14, HSP70, β-actin, and GAPDH expression. **(C)** Western blot analysis of mTORC1 pathway markers. **(D)** Western blot analysis of total eIF4E and phosphorylated eIF4E. **(E)** Schematic model illustrating how the IT-linked K219N mutation disrupts protein synthesis by shifting SLFN14 RNA cleavage activity toward type II tRNAs, leading to ribosome stalling, activation of compensatory stress pathways, and ultimately cell death. The data and the raw images underlying this Figure can be found in S1 Data and S1 Raw Images.

While working with SLFN14 inducible cells, we frequently observed cell death after prolonged SLFN14 induction. To quantify this effect, we performed MTT assays at various time points after Dox induction of SLFN14. WT SLFN14 expression led to a gradual decrease in cell viability at 12 and 24 h post-induction, whereas the K219N variant caused an earlier reduction detectable at 6 h and a significantly greater decline at 24 h, indicating that the K219N mutation exacerbates SLFN14-induced cytotoxicity (Fig 4A). This is also evident from time-course analysis of protein expression by western blot. WT SLFN14 levels steadily increased after Dox induction, while housekeeping proteins (HSP70, β-actin, and GAPDH) remained relatively stable. In contrast, induction of K219N for 24 h resulted in reduced β-actin and GAPDH levels, consistent with translational inhibition and cell loss (Fig 4B).

A previous study suggested that SLFN14 activates the mTORC1 pathway as a compensatory response to translational dysregulation [3]. Activation of mTORC1 led to S6K and 4E-BP1 phosphorylation, and 4E-BP1 phosphorylation in turn relieves inhibition of eIF4E, a key cap-binding initiation factor. We found S6K and 4E-BP1 became phosphorylated within 6 hours of SLFN14 induction, indicating activation of the mTORC1 pathway (Fig 4C). However, the magnitude of activation did not differ significantly between WT and K219N lines.

Beyond mTORC1 regulation, eIF4E activity is also regulated by phosphorylation to stimulate translation of some stress-responsive mRNAs [19]. Phosphorylated eIF4E was detected after 24 hours of WT SLFN14 induction, whereas in K219N cells, phosphorylation appeared earlier and progressively increased, suggesting that IT-linked SLFN14 mutations enhanced eIF4E phosphorylation as a stress response (Fig 4D). These results suggest that, in addition to mTORC1 activation by SLFN14, MAPK pathway-driven eIF4E phosphorylation represents an additional compensatory stress response to the more severe translational arrest elicited by the K219N mutant (Fig 4E).

### Overexpression of type II tRNAs counteracts SLFN14-mediated antiviral activity

To assess the contribution of tRNA depletion to SLFN14-mediated translational repression and antiviral activity, we co-expressed individual tRNAs in SLFN14-expressing cells and assessed VACV replication. VACV replication was quantified by both the percentage of cells expressing virus-encoded GFP and its MFI. Co-expression of any of the four tested tRNA modestly increased the percentage of GFP-positive cells in SLFN14-expressing cultures (S3A and S3C Fig). Furthermore, two type II tRNA (tRNA-Leu^TAA^ and tRNA-Ser^GCT^) also significantly increased GFP intensity in cells expressing the K219N mutant (S3B and S3D Fig), indicating higher levels of viral protein synthesis. These results indicate that depletion of type II tRNAs is particularly critical for K219N-mediated translational repression and antiviral activity.

## Discussion

Our study identifies impaired translation as a major cellular effect of IT-linked SLFN14 variants. Although these mutations were previously known to reduce SLFN14 protein expression, the underlying mechanism had remained unclear and was previously attributed to a dominant-negative effect on WT protein stability. Here, we demonstrate that reduced SLFN14 protein levels reflect enhanced translational repression by IT-linked variants, with relative potency in the order K218E ≈ K219N > R223W > WT > V220D. Apart from V220D, which was reported to behave differently from other IT-linked variants [5], the extent of translational inhibition by the variants roughly correlates with disease severity: patients carrying K218E or K219N mutations had similarly low platelet counts and severe bleeding [6], whereas those carrying R223W exhibit milder symptoms. This supports the idea that translational inhibition is an important driver of platelet dysfunction in SLFN14-linked IT.

We further identify enhanced type II tRNA cleavage as the molecular mechanism underlying the stronger translational block caused by IT-linked variants. SLFN14 is known to bind ribosomes and cleave rRNA, but there are conflicting reports on whether IT-linked mutations reduce or enhance rRNA degradation [3,5]. Through detailed time-course studies, we found that the K219N variant decreases rRNA cleavage while enhancing tRNA cleavage in HEK293T cells. Notably, at earlier time points when translational inhibition became prominent, type II tRNAs were almost completely depleted by the K219N variant, whereas rRNA degradation remained minimal. Moreover, the unbiased ribo-seq analysis revealed codon-specific ribosome stalling consistent with depletion of type II tRNAs. We therefore propose that physiological SLFN14 activity involves cleavage of both rRNA and tRNA, but that the K219N mutation impairs rRNA cleavage to a greater extent than tRNA cleavage, thereby shifting its activity towards tRNA cleavage. Basal expression levels of SLFN14 in megakaryocytes or platelets are presumably not high enough to substantially cleave tRNA or rRNA and impair translation, whereas IT-linked variants could enhance tRNA depletion even at these levels, leading to ribosome stalling, stress responses, and cell death. Consistent with a previous report [3], we found that SLFN14 activated the mTORC1 pathway. However, while K219N did not further enhance mTORC1 activation, it enhanced eIF4E phosphorylation, known to promote translation of growth-promoting and stress-responsive mRNAs [19], suggesting an adaptive attempt to compensate for global translational repression. How the K219N mutation alters RNA substrate specificity will require further structural and functional studies. Interestingly, an AlphaFold3 [20] model of SLFN14 in complex with tRNA-Leu^TAA^ positions K219 near the tRNA acceptor stem, where cleavage is predicted to occur (S4 Fig).

Our findings also suggest that tRNA depletion is a major antiviral mechanism of SLFN14. SLFN14 has been reported to cleave viral genomic RNAs in vitro [11,12]. Here, we show that it can also effectively inhibit cytoplasmic DNA viruses. The enhanced antiviral activity of IT-linked variants underscores a direct correlation between tRNA cleavage, translational arrest, and viral restriction. By modulating codon-dependent translation through targeted depletion of type II tRNAs, interferon-induced SLFN14 may provide broad-spectrum antiviral activity, since all viruses depend on the host translational machinery.

The preferential targeting of type II tRNAs by SLFN14 suggests that this is a conserved regulatory strategy within the SLFN family. Although the RNase domains of SLFN11 and SLFN12 display broad RNA substrate specificity in vitro, the full-length proteins selectively target type II tRNAs, particularly tRNA-Leu^TAA^, in cells [21–23]. SLFN14 likewise has broad RNA substrate specificity in vitro and is known to target rRNA in cells, but here we show that it preferentially depletes type II tRNAs in HEK293T cells, indicating that type II tRNAs are a common target for SLFN proteins. SLFN11 is activated in response to DNA damage [22], whereas SLFN12 can be activated by small-molecule “molecular glues” [21]. In contrast, SLFN14 does not appear to require any activation signal beyond transcriptional induction.

A limitation of our current study is that all experiments were performed in HEK293T cells and none in megakaryocytes. Studies in HEK293T cells have previously played an important role in defining the molecular function of SLFN14 [5], and this system was chosen here to compare the effects of IT-associated mutations across a wider range of expression levels. However, SLFN14 is constitutively expressed primarily in megakaryocytes and erythroid precursors and is induced in other cell types only under interferon-stimulated conditions. Accordingly, the implications of the SLFN14 functions revealed here for IT pathogenesis should be interpreted with some caution. In a previous study, platelets derived from some IT patients and an immortalized megakaryocyte cell line expressing SLFN14^K219N^ showed increased rRNA degradation [3], although tRNA levels were not examined. By contrast, our studies unexpectedly show that SLFN14^K219N^ is associated with reduced rRNA cleavage and enhanced type II tRNA cleavage in HEK293T cells. It is therefore possible that the extent of rRNA cleavage by SLFN14^K219N^ differs across cell types and experimental conditions. We consider the identification of selective targeting of type II tRNAs by IT-associated SLFN14 variants to be a key contribution of this study and propose that this effect, together with dysregulated rRNA degradation, contributes to pathogenesis. Future studies in megakaryocytes will be important to validate this idea.

## Materials and methods

### Reagents

The antibodies against the following proteins were used in the immunoblotting: mCherry (Bio-techne, NBP2-25157), HSP70 (Santa Cruz, sc-137210), β-actin (Sigma, A2228), GAPDH (Cell Signaling Technology, #2118), 4E-BP1 (Cell Signaling Technology, #9452), Phospho-4E-BP1(Thr37/46) (Cell Signaling Technology, #2855), eIF4E (Cell Signaling Technology, #9742), Phospho-eIF4E (Ser209) (Cell Signaling Technology, #9741), p70 S6 Kinase (Cell Signaling Technology, #9202), Phospho-p70 S6 Kinase (Cell Signaling Technology, #9205). Additional reagents included high-range RNA ladder (ThermoFisher, SM1821) and the Low Range ssRNA Ladder (NEB, N0364S).

### Cells and viruses

Human embryonic kidney (HEK) 293T cells (ATCC CRL-3216) were cultured in Dulbecco’s Modified Eagle Medium (DMEM) supplemented with 10% fetal bovine serum (FBS) at 37 °C with 5.0% CO_2_. WT vaccinia virus (VACV) strain Western Reserve (WR) and the GFP-expressing VACV K1/C7 deletion mutant (vK1^−^C7^−^/GFP^+^) were described previously [19–21].

### Plasmid construction

Plasmids were constructed using PCR products or synthesized DNA fragments using the NEBuilder HiFi DNA Assembly Kit (NEB). pcDNA6.2/mCherry-p2A-hSLFN14 was constructed with DNA fragments synthesized by Twist Bioscience. Point mutations (K218E, K219N, V220D, R223W) were introduced using PCR products from synthesized oligos containing the desired mutations. mCherry-tagged SLFN14 WT and K219N sequences were PCR-amplified and cloned into a modified piggyBac vector with a Dox-inducible promoter and the reverse tetracycline transactivator [24] (Gift of Satoshi Narumi). Plasmids expressing tRNA-Leu^CAA^ and tRNA-Phe^GAA^ were described previously [25]. Plasmids expressing tRNA-Leu^TAA^ and tRNA-Ser^GCT^ were assembled using synthesized DNA fragments from Integrated DNA Technologies (IDT). All constructs were verified by whole-plasmid sequencing (Plasmidsaurus). Primer sequences are listed in S1 Table. pcDNA6.2/mCherry-p2A-hSLFN14 sequence is provided as S3 Data file.

### Generation of inducible SLFN14 cell lines

293T parental cells were transfected with 500 ng of the piggyBac vector encoding SLFN14 and 200 ng transposase expression plasmid (System Biosciences) in 6-well plates. After 72 h, cells were replated and selected with blasticidin (15 μg/mL) for 3 days, followed by single-cell sorting into 96-well plates. After 2 weeks, clones were screened for doxycycline (2 μg/mL) inducibility by mCherry expression. Positive clones were expanded, and SLFN14 expression was validated by immunoblotting with anti-mCherry antibody.

### Antiviral assays with SLFN14 variant

For transient-expression assays, HEK293T cells were transfected with plasmids expressing WT or mutant mCherry-SLFN14. After 24 h, cells were infected with vK1^−^C7^−^/GFP^+^ VACV for 15 h. GFP expression was analyzed by fluorescence microscopy and quantified by flow cytometry, as described [26]. mCherry and GFP were used to identify and stratify cells according to SLFN14 expression or infection status, and mean fluorescence intensities measured across large cell populations were used as a qualitative readout.

### Antiviral assays in inducible cell lines

WT or K219N SLFN14-inducible 293T cells were seeded in 24-well plates and left untreated or induced with doxycycline (2 μg/mL) for 24 h. Cells were infected with VACV (MOI 0.5) for 2 h at room temperature. After adsorption, cells were washed twice with PBS and incubated at 37 °C. After 0, 24, and 48 h, samples were collected, and viral titers were determined by plaque assay on CV-1 cells. In some experiments, cells were also analyzed by flow cytometry.

### Assay of nascent protein synthesis

Nascent protein synthesis was measured using the Click-iT Plus OPP Alexa Fluor 488 Protein Synthesis Assay Kit (Thermo Fisher Scientific). Cells were pulsed with O-propargyl-puromycin (OPP) for 30 min, followed by the Click-iT reaction and analysis by flow cytometry (BD LSR-II)*.*

### Viral rescue assay with co-transfection SLFN14 and exogenous tRNA

The experiment was performed essentially as described previously in our study of the tRNA-Phe targeting endoribonuclease SAMD9 [25]. Briefly, HEK293T cells were co-transfected with plasmids expressing SLFN14 mCherry fusion plasmids and a plasmid expressing tRNA-Phe^GAA^, tRNA-Leu^CAA^, tRNA-Leu^TAA^, or tRNA-Ser^GCT^ (pcDNA3.1 as control) for 12 h. Cells were then infected with VACV/GFP+ for 16 h, and infection rates were determined by flow cytometry.

### RNA analysis: rRNA and tRNA

Total RNA was extracted using TRIzol reagent. For rRNA analysis, 0.5 μg of RNA was resolved on a 2% agarose gel. For tRNA analysis, 2.5 μg of RNA was separated by 10% denaturing urea-PAGE and visualized with SYBR Gold staining.

### MTT assay

Cell viability of SLFN14 inducible 293T cells was tested using the MTT assay. Briefly, cells were seeded in 96-well plates and left untreated or treated with doxycycline (2 μg/mL) for indicated times (6, 12, or 24 h). Ten µL of MTT reagent (Invitrogen, V13154) was added to each well and incubated for 4 h at 37 °C. The reaction was terminated by adding 100 μL of DMSO, followed by a 10 min incubation at 37 °C. Absorbance was measured at 540 nm using a microplate reader (BioTek).

### Immunoblotting

Cell lysates were separated on 4%–12% SDS-PAGE gradient gels (Bio-Rad) and transferred to nitrocellulose membranes (Thermo Fisher Scientific). Membranes were blocked with TBS containing 5% nonfat dry milk and 0.1% Tween-20 for 1 h at room temperature, incubated overnight at 4 °C with primary antibodies, and then probed with HRP-conjugated secondary antibodies. Bands were visualized by enhanced chemiluminescence (ECL-Plus, GE Healthcare). For all conditions within the same experiment, equal numbers of cells were plated at the start of the experiment. Up to three housekeeping proteins (HSP70, β-actin, and GAPDH) were examined, as different proteins may be affected differently by global translational repression mediated by SLFN14, in part depending on their cellular half-lives. Signals from these housekeeping proteins were interpreted as supportive rather than definitive normalization controls.

### Northern blotting

tRNAs were analyzed as described previously [23]. Briefly, RNA samples were resolved on 15% TBE-urea gels, transferred to positively charged nylon membranes, and cross-linked by UV. DIG-labeled oligonucleotide probes specific for individual tRNAs (S1 Table) were hybridized to the membranes. Probes were generated using the DIG Oligonucleotide 3′-End Labeling Kit (Roche). Detection was performed using CDP-Star substrate (Roche) and chemiluminescence.

### Ribosome profiling (Ribo-seq)

WT or K219N SLFN14-inducible 293T cells were seeded in 6-well plates and left untreated or induced with doxycycline (2 μg/mL) for 24 h. The 6-well plates were pre-coated with poly-D-lysine (Gibco) for 5 min at room temperature to enhance the cell attachment. Following treatment, cells were washed once with ice-cold PBS and lysed in polysome lysis buffer (10 mM HEPES, pH 7.4, 5 mM MgCl_2_, 100 mM KCl, 1% Triton X-100, 100 μg/mL cycloheximide). Lysates were clarified by centrifugation at maximum speed (≥13,000*g*) for 15 min at 4 °C, and the supernatant was collected for downstream ribo-seq analysis. Ribo-seq libraries were constructed by modifying the Ezra-seq method, as previously described [27]. In brief, the whole cell lysates were digested with E. coli RNase I (Ambion, 750 U per 100 A260 units) by agitation at 4 °C for 1 h. RNAs were extracted using Trizol LS reagent (Invitrogen) followed by isopropanol precipitation. The ribosome-protected mRNA fragments (RPFs) were separated on a 15% polyacrylamide TBE-urea gel (Invitrogen) and visualized using SYBR Gold (Invitrogen). Selected regions in the gel corresponding to 25–35 nt were excised and dissolved by soaking in 400 μl RNA elution buffer (300 mM NaOAc pH 5.2, 1 mM EDTA, 0.1 U/μl SUPERase·In (Invitrogen)) for 10 min at 70 °C. The gel debris was removed using a Spin-X column (Corning), followed by isopropanol precipitation. 4 μl RNAs (10–200 ng) were mixed with 1 nmol ATP, 1 μl T4 PNK (NEB), 20 U SUPERase·In, 1 μl Poly(A) Polymerase (NEB), and 1 μl homemade Ezra enzyme in 1 × T4 PNK buffer and incubated at 37 °C for 30 min followed by 70 °C for 10 min. Then, ligation was performed for 90 min at 25 °C by mixing with a 10 μl reaction mixture (1 pmol biotinylated 5′ end adaptor, 1 × T4 Rnl2 reaction buffer, 20 U SUPERase·In, 12.5% PEG8000, and 200 U T4 RNA ligase 2 truncated KQ (NEB)). The ligated RNA samples were pulled down after incubation with 20 uL pre-washed streptavidin magnetic beads (NEB) at room temperature for 10 min. After washing once with 100 μL 2 × SSC (Invitrogen), beads were resuspended in 12 μl nuclease-free water and mixed with 8 μl cDNA synthesis mixture (1 poml RT primer, 1 × first strand buffer, 100 nmol DTT, 1 nmol dNTP, 20 U SUPERase·In and 100 ng m-MLV) followed by incubation at 50 °C for 20 min. After washing once with 2 × SSC, beads were resuspended in 20 μl nuclease-free water and incubated at 95 °C for 2 min, then immediately placed on the ice for 1 min. After placing on magnet stand for 1 min, the supernatant cDNA was amplified by PCR using barcoded sequencing primers. PCR was performed by mixing 1 × HF buffer, 0.5 mM dNTP, 0.25 μM PCR primers and 0.025 U Phusion polymerase. PCR was carried out under the following conditions: 98 °C, 30 s; (98 °C, 5 s; 67 °C, 15 s; 72 °C, 20 s) for 13 cycles; 72 °C, 2 min. PCR products were separated on an 8% polyacrylamide TBE gel (Invitrogen). DNA products with the expected size 170 bp were excised and recovered from DNA elution buffer (300 mM NaCl, 1 mM EDTA). After quantification by Qubit 4 Fluorometer (Invitrogen), equal amounts of barcoded samples were pooled and sequenced using MiniSeq (Illumina). The oligonucleotide sequences are listed in S1 Table.

### Ribo-seq analysis

To align sequencing reads, the 5′ and 3′ adapters of the reads were trimmed by Cutadapt (version 2.8) [28]. The trimmed reads with length shorter than 15 nucleotides were excluded from the analysis. To keep accurate reading frame of Ribo-seq, low-quality bases at both ends of the reads were not subject to clip. The trimmed reads were first aligned to rRNAs using Bowtie (version 1.2.3) [29]. The rRNA sequences were downloaded from the nucleotide database of NCBI and RNAcentral. The reads unaligned to rRNAs were then mapped to the custom human transcriptome using STAR (version 2.7.10a) [30]. To avoid ambiguity, reads mapped to multiple positions or with >2 mismatches were disregarded for further analysis. The custom transcriptome was generated based on the reference genome and annotations obtained from Ensembl using human release 109 (GRCh38.p14). Protein-coding genes were extracted, and a single transcript was selected for each gene on the following procedure. For each gene, the transcript with the highest APPRIS score was initially selected. If the selected transcripts have equal APPRIS scores, the transcript with the longest coding sequence (CDS) was included in the custom transcriptome. Mapping to ribosomal P sites was performed by shifting the read position from the 5′ ends to the position by 12 nt.

For codon occupancy analysis, ribosome density at each codon was calculated by normalizing the read count with the average read counts per codon of the transcript, and then was averaged across the transcriptome. The subsequence abundance shift plots were created following diricore analysis [31].

### Quantification and statistical analysis

Data were analyzed using GraphPad Prism 9. Statistical methods, including one-way ANOVA, are specified in the figure legends.

## Supporting information

S1 Fig RNase activity is required for SLFN14 to inhibit global protein synthesis and viral replication. **(A)** Multiple sequence alignment of human SLFN proteins. The numbering above the alignment indicates positions in SLFN14. Note SLFN14 E206 is a catalytic residue conserved in human SLFNs. **(B)** HEK293T cells were transfected with mCherry-P2A-SLFN14 variants. Nascent protein synthesis was measured by OPP incorporation as described in Fig 1. Representative flow cytometry plots show OPP levels relative to mCherry expression. Quantification is shown in **(F)**. **(C)** HEK293T cells were transfected with mCherry-P2A-SLFN14 variants and infected with VACV/GFP^+^ as described in Fig 1. Representative flow cytometry plots show GFP expression in SLFN14-expressing versus untransfected cells. Quantification is shown in **(E)**. **(D)** HEK293T cells were co-transfected with a plasmid expressing mCherry together with a plasmid expressing N-terminally Flag-tagged SLFN14 or pcDNA3 as a vector control. At 16 h post-transfection, nascent protein synthesis was measured by OPP incorporation for 30 min. Representative flow cytometry plots show OPP levels relative to mCherry expression, with mCherry marking cotransfected cells. The vertical dashed line indicates the mean fluorescence intensity of the cell populations. Quantification is shown in G. Statistical significance was assessed by pairwise Student’s *t* tests (**** *P* < 0.0001). The data underlying this Figure can be found in S1 Data.
(TIF)

S2 Fig SLFN14 inhibits global protein synthesis and viral replication, effects exacerbated by IT-associated mutations. **(A, B)** Dox-inducible 293T cell lines expressing mCherry-SLFN14 (WT or K219N) were left untreated or induced for the indicated times, and protein synthesis was measured as in Fig 1. Representative plots are shown in **(A)**, with quantification of relative OPP MFI in Dox+ versus Dox− conditions in **(B)**. **(C–F)** Dox-inducible 293T cell lines expressing mCherry-SLFN14 (WT or K219N) were left untreated or induced with Dox and subsequently infected with VACV/GFP^+^. Representative flow cytometry plots **(C)** and quantification of infection rates (GFP^+^ cells, 1 = 100%) **(D)**. **(E)** Viral titers at 0, 24, and 48 h post-infection were measured by plaque assay on Vero cells. Statistical analysis was performed using one-way ANOVA (***P < 0.001, ****P < 0.0001). **(F)** Western blot analysis of mCherry-SLFN14 expression in inducible cell lines using anti-mCherry and anti-HSP70 antibodies. “+Dox” indicates induction with doxycycline for 24 h. “+virus” indicates infection with VACV for 12 h. Statistical analysis was performed using one-way ANOVA (****P < 0.0001). The data and the raw images underlying this Figure can be found in S1 Data and S1 Raw Images.
(TIF)

S3 Fig Overexpression of type II tRNAs counteracts the antiviral effect of SLFN14. HEK293T cells were transfected with mCherry-P2A-SLFN14 (WT or K219N) together with the indicated tRNA expression constructs for 8 h and then infected with VACV/GFP^+^ for 15 h. **(A)** Representative flow cytometry plots. **(B)** Histograms of GFP fluorescence intensity from the infected cell populations. **(C)** Quantification of the percentage of GFP⁺ cells shown in (A). **(D)** Quantification of GFP MFI in infected cell populations shown in (B). The data underlying this Figure can be found in S1 Data.
(TIF)

S4 Fig AlphaFold3 model of SLFN14 in complex with tRNA-Leu^TAA^. A complex of the SLFN14 dimer and tRNA-Leu^TAA^ was modeled using AlphaFold3, with an ipTM score of 0.74 and a pTM score of 0.77. The entire model is colored according to local confidence metrics (pLDDT). An enlarged view of the IT-associated residues relative to the bound type II tRNA is shown on the right, with the tRNA variable stem-loop indicated. The data underlying this Figure can be found in S2 Data.
(TIF)

S1 Table Oligonucleotides used in this study. (DOCX)

S1 Data Numeric values of graphs in all the figures. (XLSX)

S2 Data AlphaFold3 model of SLFN14 in complex with tRNA-Leu^TAA^. (ZIP)

S3 Data Nucleotide sequence of the mCherry-P2A-SLFN14 plasmid in FASTA format. (FASTA)

S1 Raw Images Original gel images for figures. (PDF)

## Abbreviations

CDS: coding sequence
CHX: cycloheximide
DMEM: Dulbecco’s Modified Eagle Medium
Dox: doxycycline
FBS: fetal bovine serum
IT: inherited thrombocytopenia
MFI: mean fluorescence intensity
MK: megakaryocyte
OPP: O-propargyl-puromycin
SLFNs: Schlafens
VACV: vaccinia‌‌ virus
WT: wild-type
