# Supplementary figures and images for: Type II tRNA cleavage by SLFN14 endoribonuclease variants linked to inherited thrombocytopenia drives global translational repression
Source: PLoS Biol. 2026 May 29;24(5):e3003830. doi: 10.1371/journal.pbio.3003830 (PMC13245857; doi:10.1371/journal.pbio.3003830)

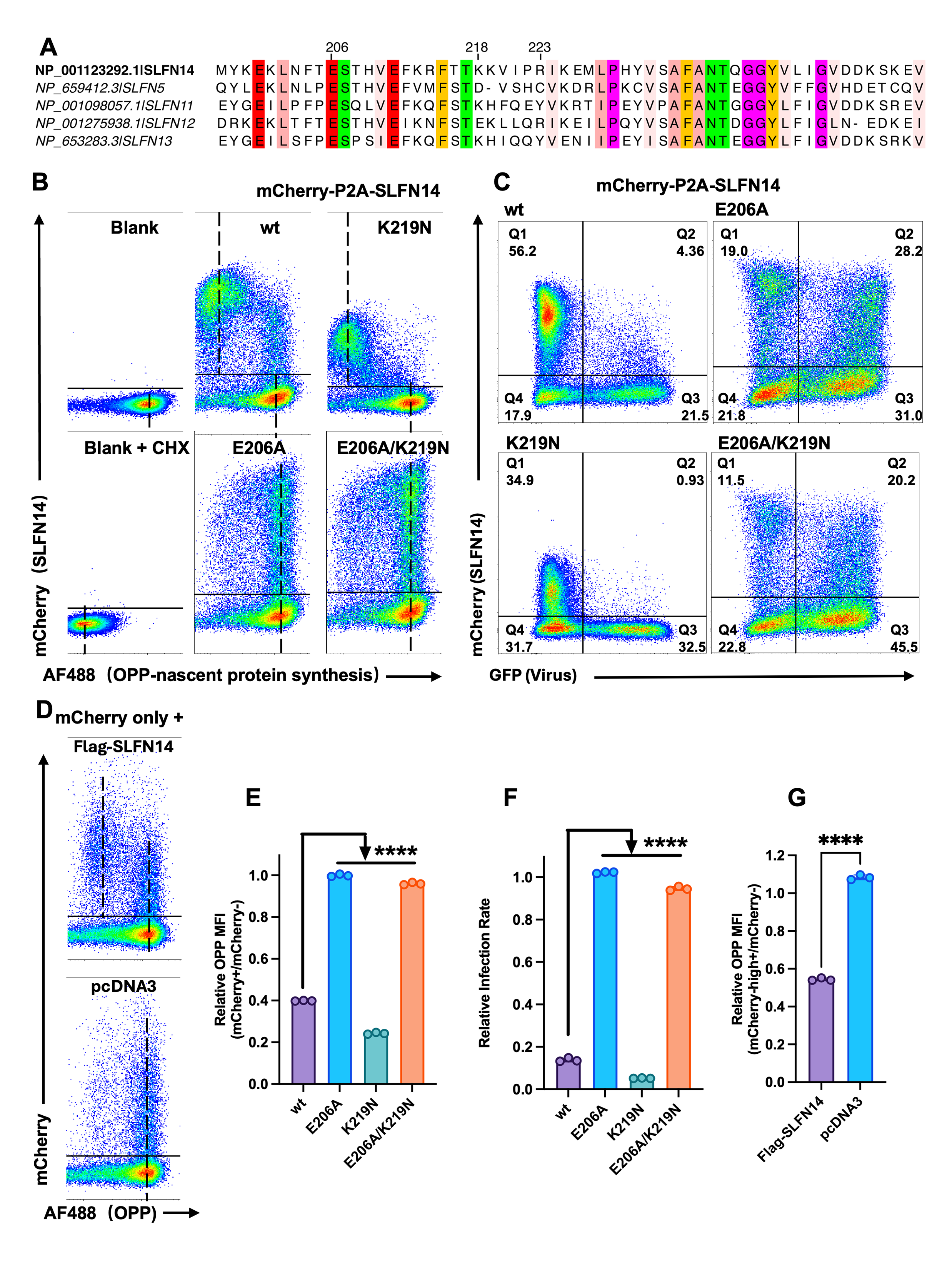

Supplement: S1 Fig — (A) Multiple sequence alignment of human SLFN proteins. The numbering above the alignment indicates positions in SLFN14. Note SLFN14 E206 is a catalytic residue conserved in human SLFNs. (B) HEK293T cells were transfected with mCherry-P2A-SLFN14 variants. Nascent protein synthesis was measured by OPP incorporation as described in Fig 1. Representative flow cytometry plots show OPP levels relative to mCherry expression. Quantification is shown in (F). (C) HEK293T cells were transfected with mCherry-P2A-SLFN14 variants and infected with VACV/GFP+ as described in Fig 1. Representative flow cytometry plots show GFP expression in SLFN14-expressing versus untransfected cells. Quantification is shown in (E). (D) HEK293T cells were co-transfected with a plasmid expressing mCherry together with a plasmid expressing N-terminally Flag-tagged SLFN14 or pcDNA3 as a vector control. At 16 h post-transfection, nascent protein synthesis was measured by OPP incorporation for 30 min. Representative flow cytometry plots show OPP levels relative to mCherry expression, with mCherry marking cotransfected cells. The vertical dashed line indicates the mean fluorescence intensity of the cell populations. Quantification is shown in G. Statistical significance was assessed by pairwise Student’s t tests (**** P < 0.0001). The data underlying this Figure can be found in S1 Data. (TIF) [file pbio.3003830.s001.tif]

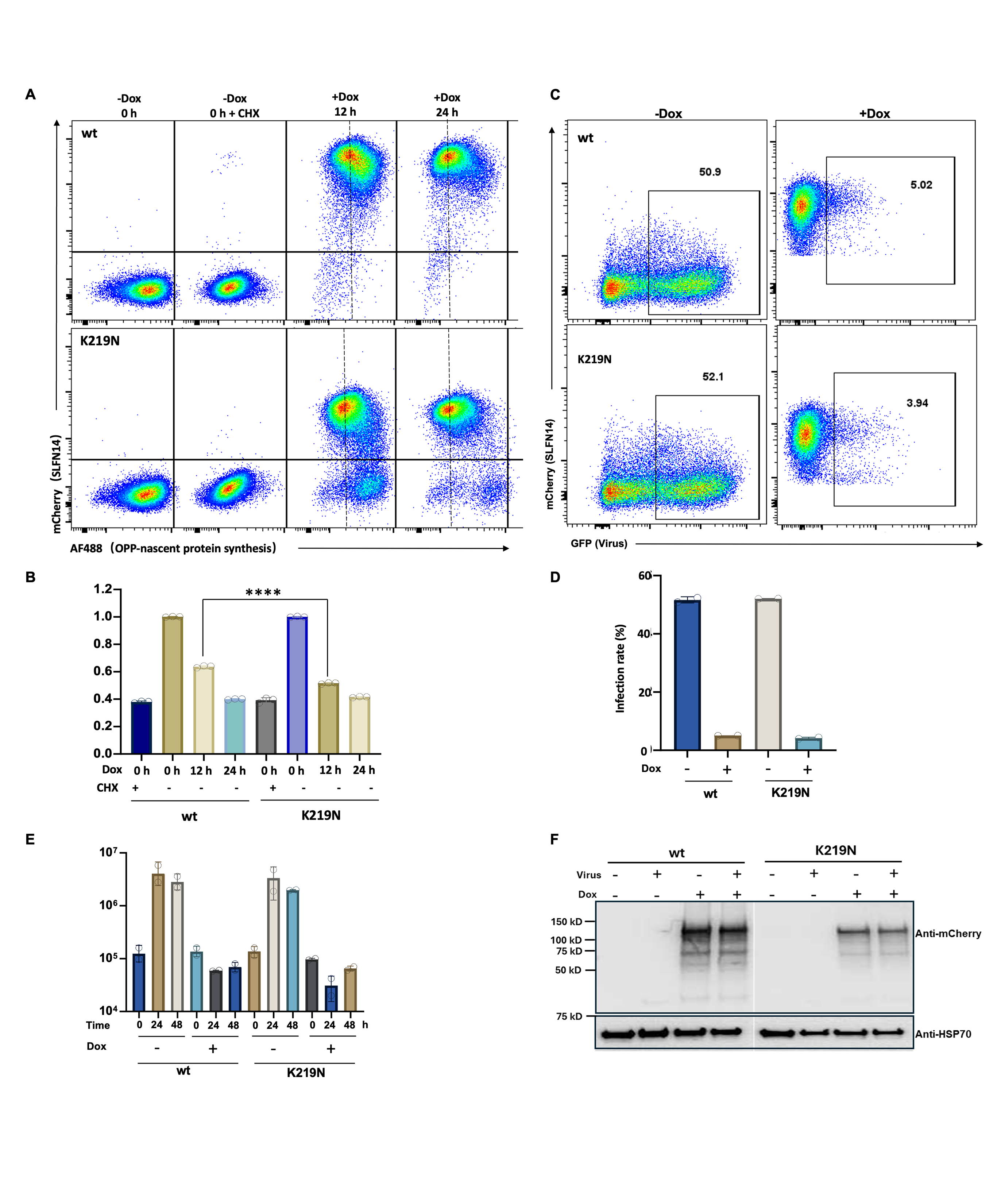

Supplement: S2 Fig — (A, B) Dox-inducible 293T cell lines expressing mCherry-SLFN14 (WT or K219N) were left untreated or induced for the indicated times, and protein synthesis was measured as in Fig 1. Representative plots are shown in (A), with quantification of relative OPP MFI in Dox+ versus Dox− conditions in (B). (C–F) Dox-inducible 293T cell lines expressing mCherry-SLFN14 (WT or K219N) were left untreated or induced with Dox and subsequently infected with VACV/GFP+. Representative flow cytometry plots (C) and quantification of infection rates (GFP+ cells, 1 = 100%) (D). (E) Viral titers at 0, 24, and 48 h post-infection were measured by plaque assay on Vero cells. Statistical analysis was performed using one-way ANOVA (***P < 0.001, ****P < 0.0001). (F) Western blot analysis of mCherry-SLFN14 expression in inducible cell lines using anti-mCherry and anti-HSP70 antibodies. “+Dox” indicates induction with doxycycline for 24 h. “+virus” indicates infection with VACV for 12 h. Statistical analysis was performed using one-way ANOVA (****P < 0.0001). The data and the raw images underlying this Figure can be found in S1 Data and S1 Raw Images. (TIF) [file pbio.3003830.s002.tif]

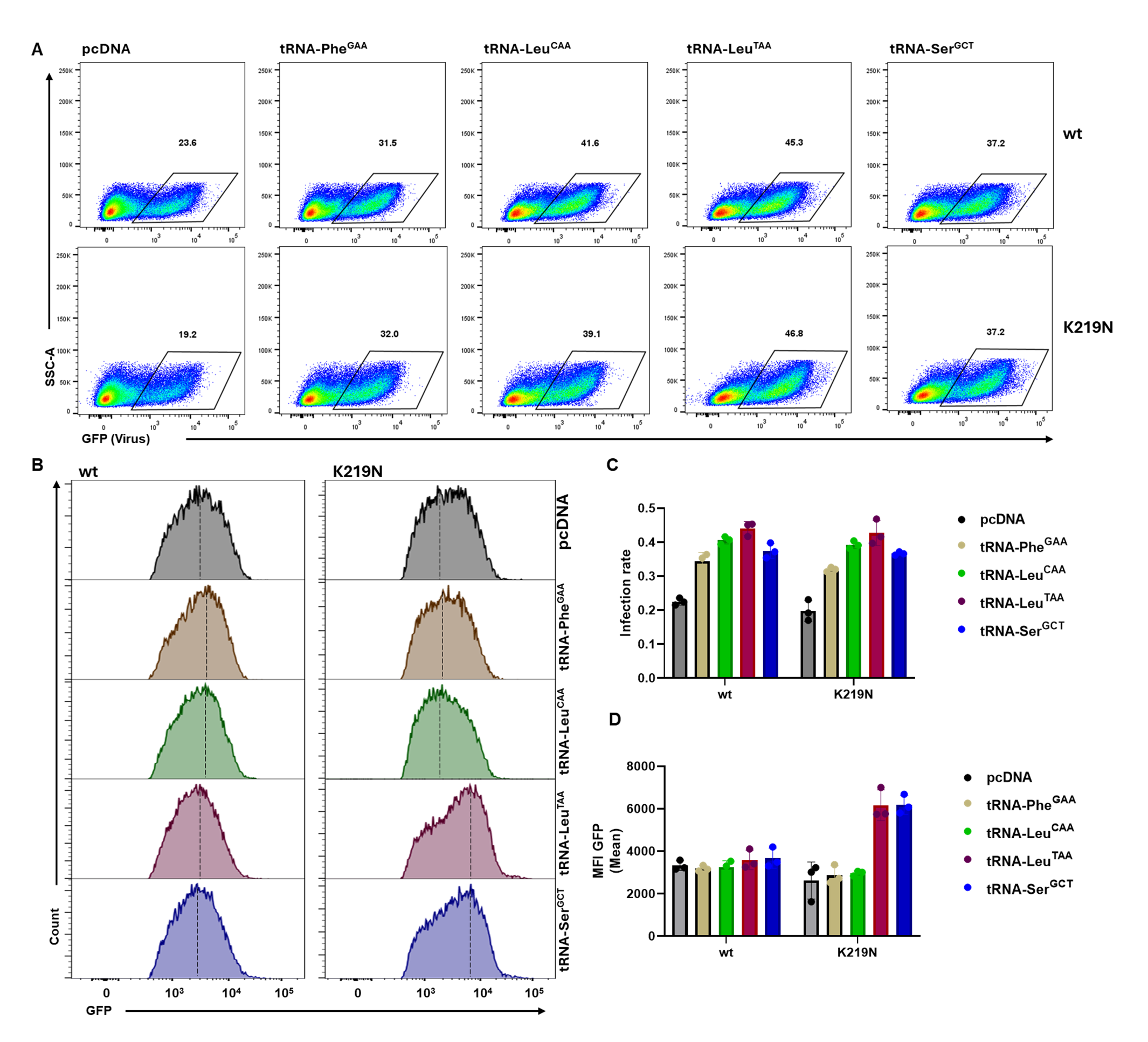

Supplement: S3 Fig — HEK293T cells were transfected with mCherry-P2A-SLFN14 (WT or K219N) together with the indicated tRNA expression constructs for 8 h and then infected with VACV/GFP+ for 15 h. (A) Representative flow cytometry plots. (B) Histograms of GFP fluorescence intensity from the infected cell populations. (C) Quantification of the percentage of GFP⁺ cells shown in (A). (D) Quantification of GFP MFI in infected cell populations shown in (B). The data underlying this Figure can be found in S1 Data. (TIF) [file pbio.3003830.s003.tif]

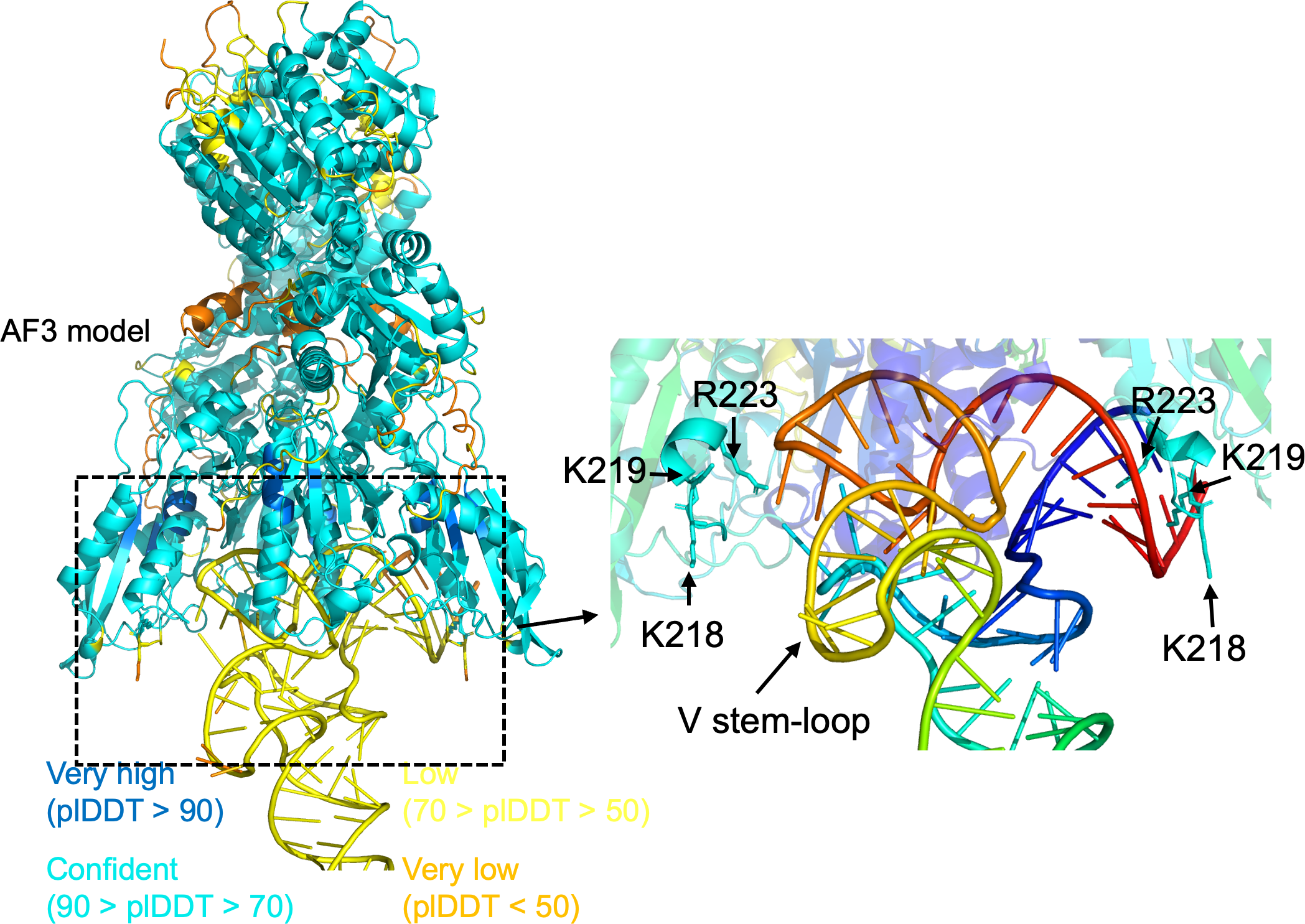

Supplement: S4 Fig — A complex of the SLFN14 dimer and tRNA-LeuTAA was modeled using AlphaFold3, with an ipTM score of 0.74 and a pTM score of 0.77. The entire model is colored according to local confidence metrics (pLDDT). An enlarged view of the IT-associated residues relative to the bound type II tRNA is shown on the right, with the tRNA variable stem-loop indicated. The data underlying this Figure can be found in S2 Data. (TIF) [file pbio.3003830.s004.tif]
