# Supplementary material for: Type II tRNA cleavage by SLFN14 endoribonuclease variants linked to inherited thrombocytopenia drives global translational repression
Source: PLoS Biol. 2026 May 29;24(5):e3003830. doi: 10.1371/journal.pbio.3003830 (PMC13245857; doi:10.1371/journal.pbio.3003830)
Supplement: S1 Table — (DOCX) [file pbio.3003830.s005.docx]

**Supplemental Table 1. Oligonucleotides used in this study.**

| **Oligo Name** | **Sequence (5’-3’)** |
| --- | --- |
| **hSLFN14 mutations cloning** | |
| K218E-F | AAGCGGTTCACAACTGAAAAGGTGATCCCCAGAATCAA |
| K218E-R | TCTGGGGATCACCTTTTCAGTTGTGAACCGCTTAAATT |
| K219N-F | CGGTTCACAACTAAAAACGTGATCCCCAGAATCAAAGA |
| K219N-R | GATTCTGGGGATCACGTTTTTAGTTGTGAACCGCTTAA |
| V220D-F | TTCACAACTAAAAAGGACATCCCCAGAATCAAAGAAAT |
| V220D-R | TTTGATTCTGGGGATGTCCTTTTTAGTTGTGAACCGCT |
| R223W-F | AAAAAGGTGATCCCCTGGATCAAAGAAATGCTGCCCCA |
| R223W-R | CAGCATTTCTTTGATCCAGGGGATCACCTTTTTAGTTG |
| E206A-F | GAACTTCACCGCGTCCACTCATGTGGAATTTAAGCG |
| E206A-R | GAGTGGACGCGGTGAAGTTCAGCTTTTCCTTG |
| **Northern blot Probes** | |
| tRNA-Phe^GAA^ | CGAAACCCGGGATCGAACCAGGGACCTTTA |
| tRNA-Leu^CAA^ | TGTCAGAAGTGGGATTCGAACCCACGCCTC |
| tRNA-Leu^TAA^ | TACCAGAAGTGGGGTTCGAACCCACGCGGA |
| tRNA-Ser^GCT^ | AACCACTCGGCCACCTCGTC |
| **Ribo-seq** | |
| biotinylated 5’ end adaptor | /5Biosg/AATGATACGGCGACCACCGAGATCTACACTCTTTCCCTACACGACGCTCTTCCGATCTrGrGrG |
| RT primer | GTGACTGGAGTTCAGACGTGTGCTCTTCCGATCTTTTTTTTTTTTTTTTVN |
| PCR forward primer | AATGATACGGCGACCACCGAGATCTACACTCTTTCCCTACACGACGCTCTTCCGATCT |
| RCR reverse primers (with barcode) | CAAGCAGAAGACGGCATACGAGAT TGACAT GTGACTGGAGTTCAGACGTGTGCTCT |
|  | CAAGCAGAAGACGGCATACGAGAT GGACGG GTGACTGGAGTTCAGACGTGTGCTCT |
|  | CAAGCAGAAGACGGCATACGAGAT CTCTAC GTGACTGGAGTTCAGACGTGTGCTCT |
|  | CAAGCAGAAGACGGCATACGAGAT GCGGAC GTGACTGGAGTTCAGACGTGTGCTCT |
