# Supplementary material for: Type II tRNA cleavage by SLFN14 endoribonuclease variants linked to inherited thrombocytopenia drives global translational repression
Source: PLoS Biol. 2026 May 29;24(5):e3003830. doi: 10.1371/journal.pbio.3003830 (PMC13245857; doi:10.1371/journal.pbio.3003830)
Supplement: S1 Raw Images — (PDF) [file pbio.3003830.s009.pdf]

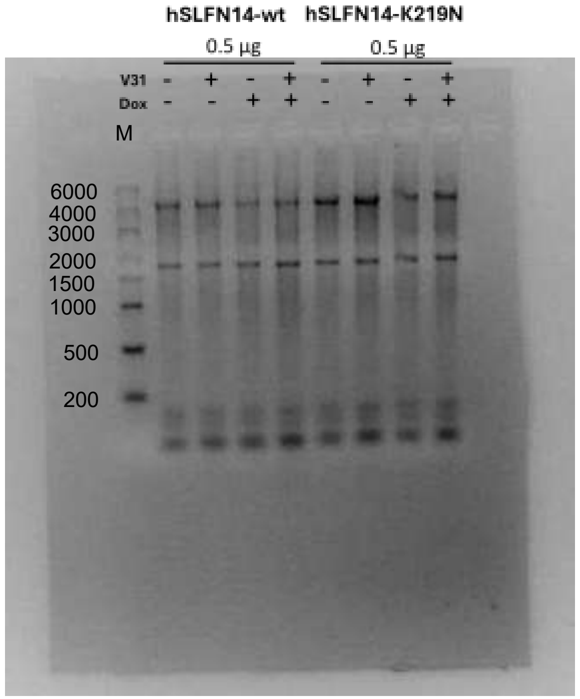

Fig 2A

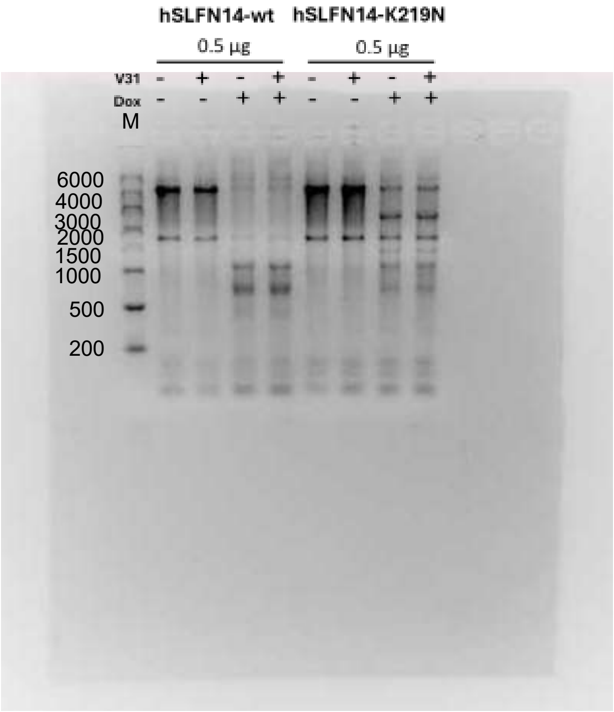

Fig 2D

2% agarose

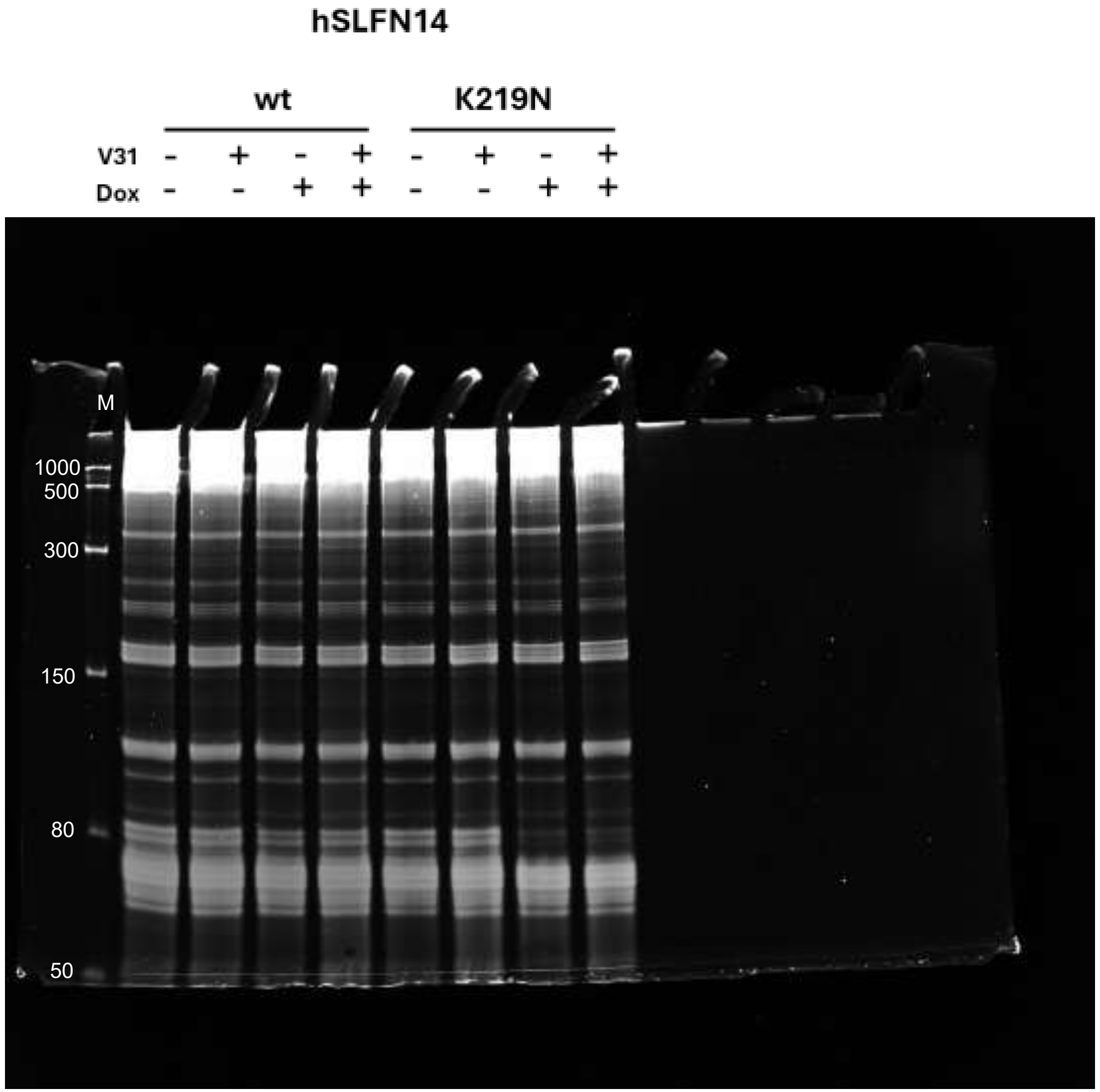

Fig 2B

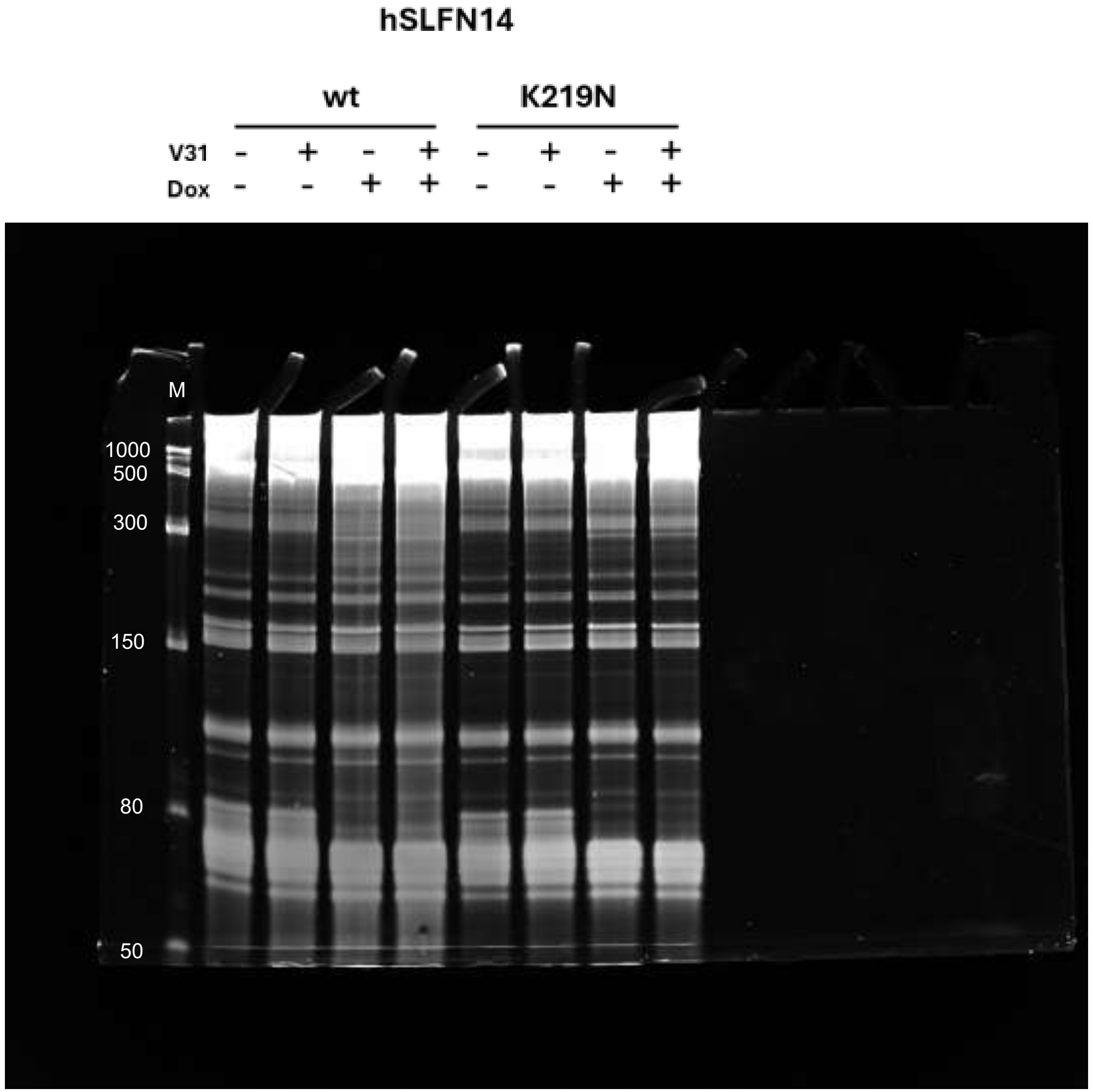

Fig 2E

10% Urea PAGE

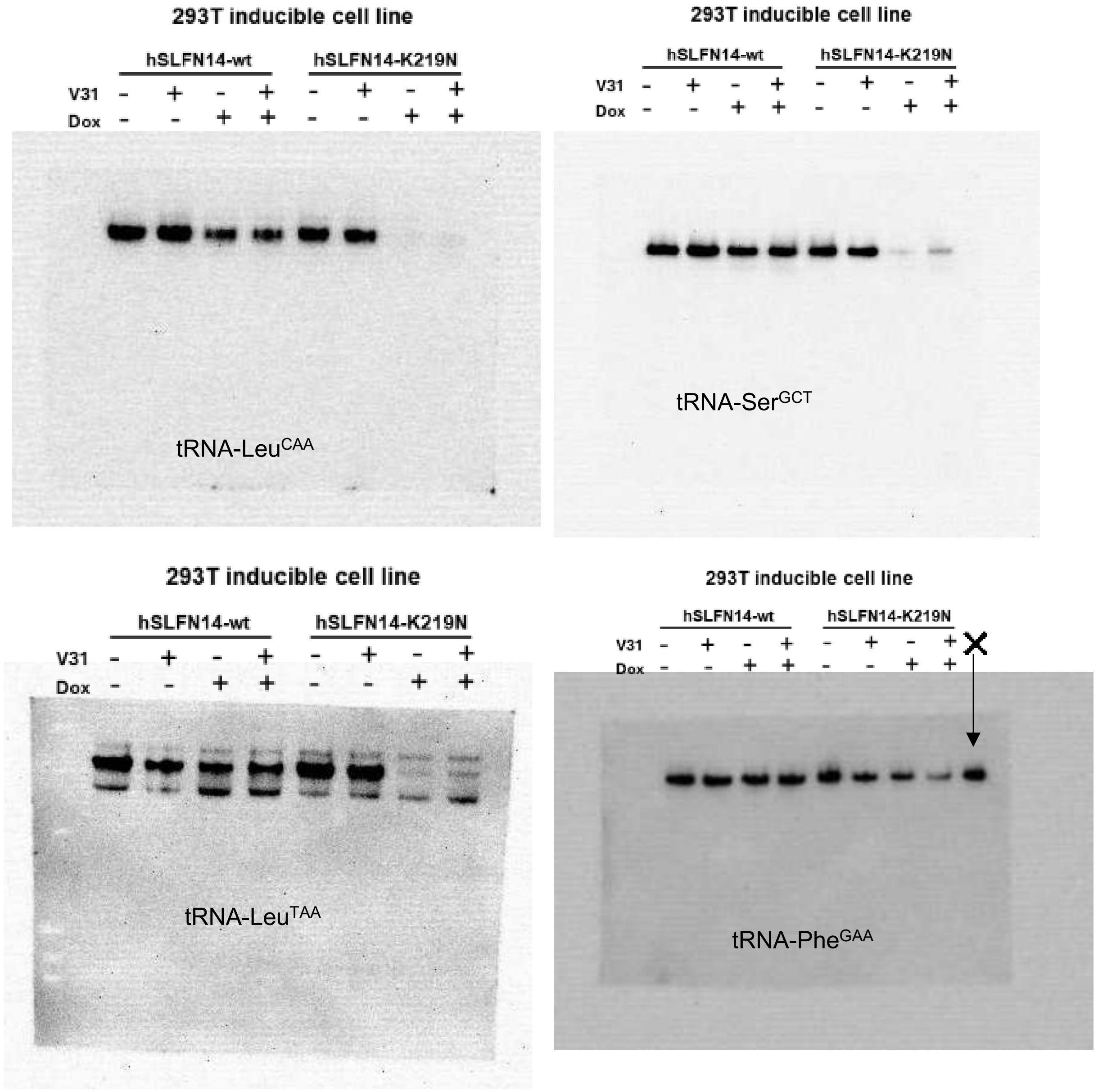

Fig 2C

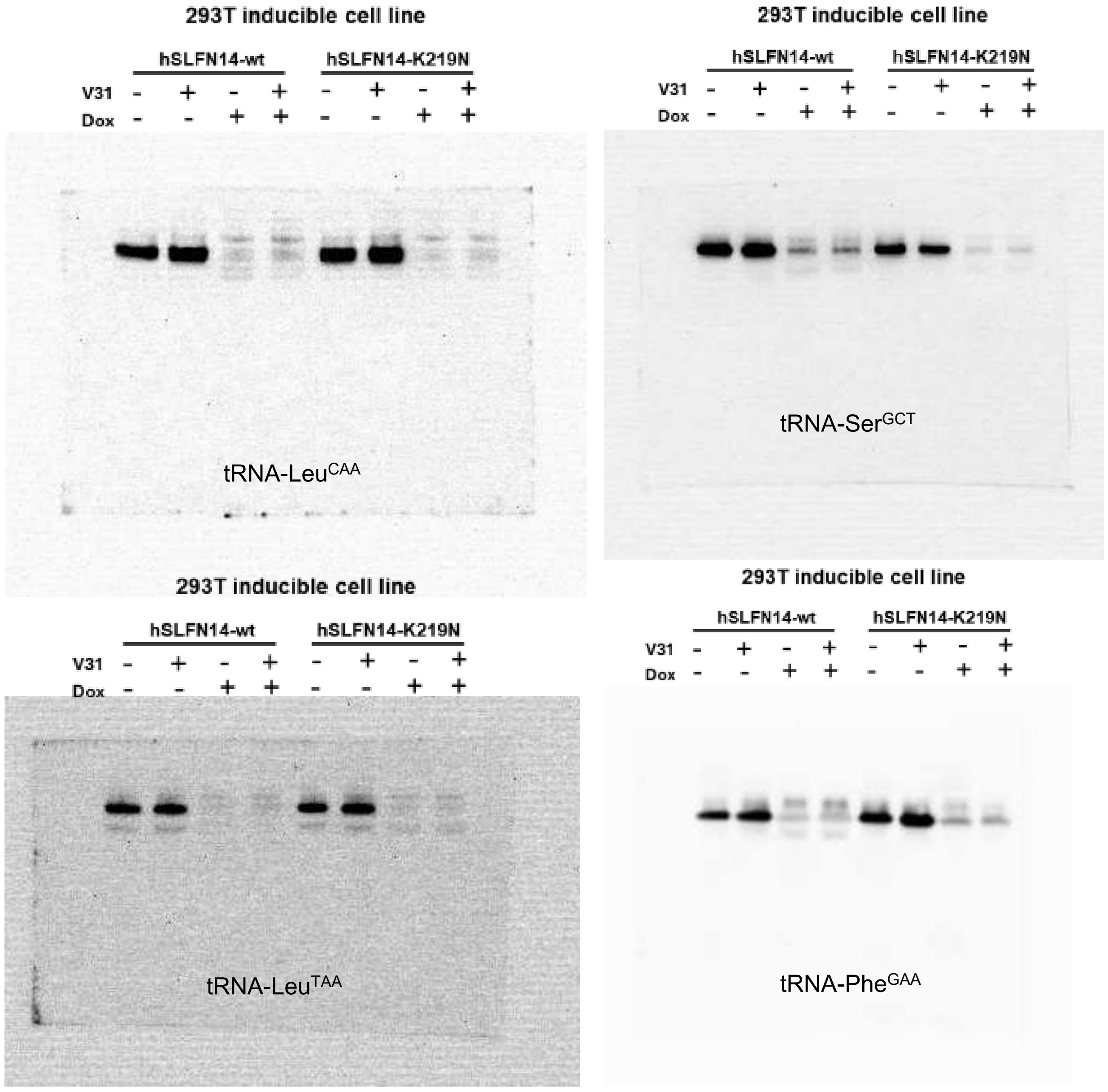

Fig 2F

Northern-blot

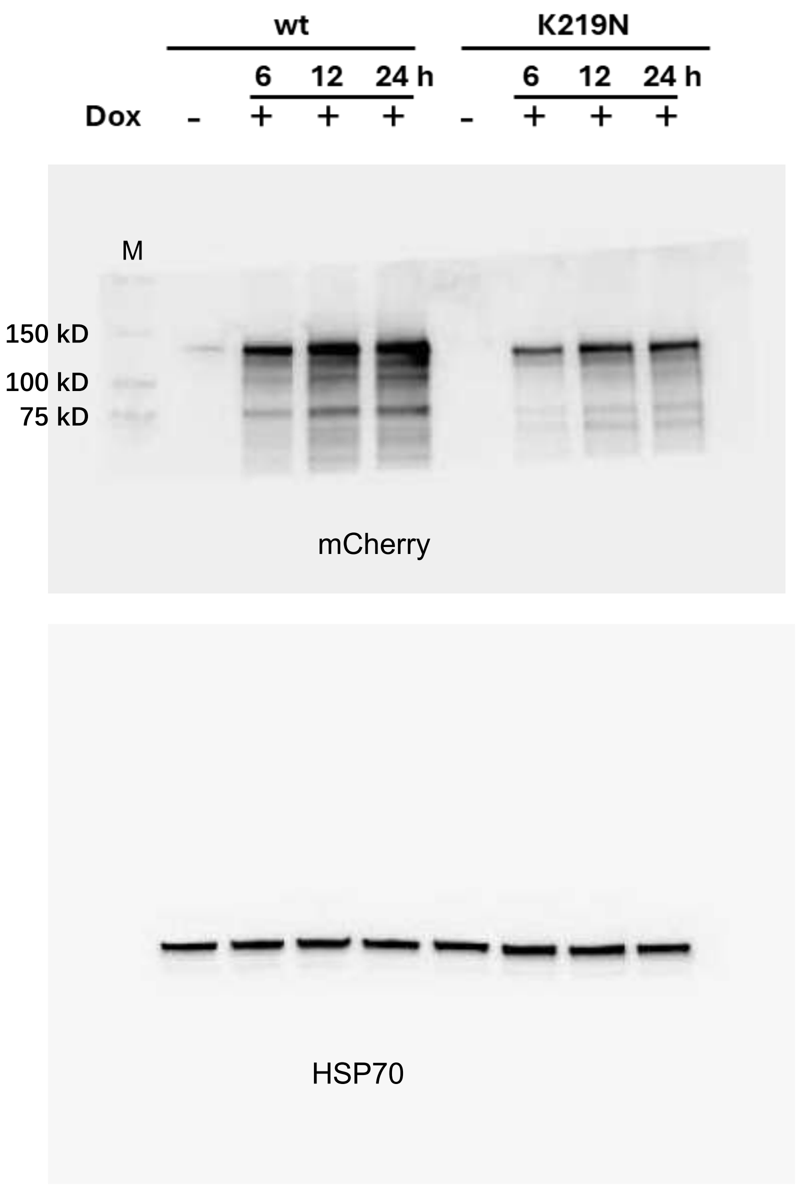
wt
K219N
6 12 24 h
6 12 24 h
Dox - + + + - + + +
M
150 kD
100 kD
75 kD
mCherry
HSP70

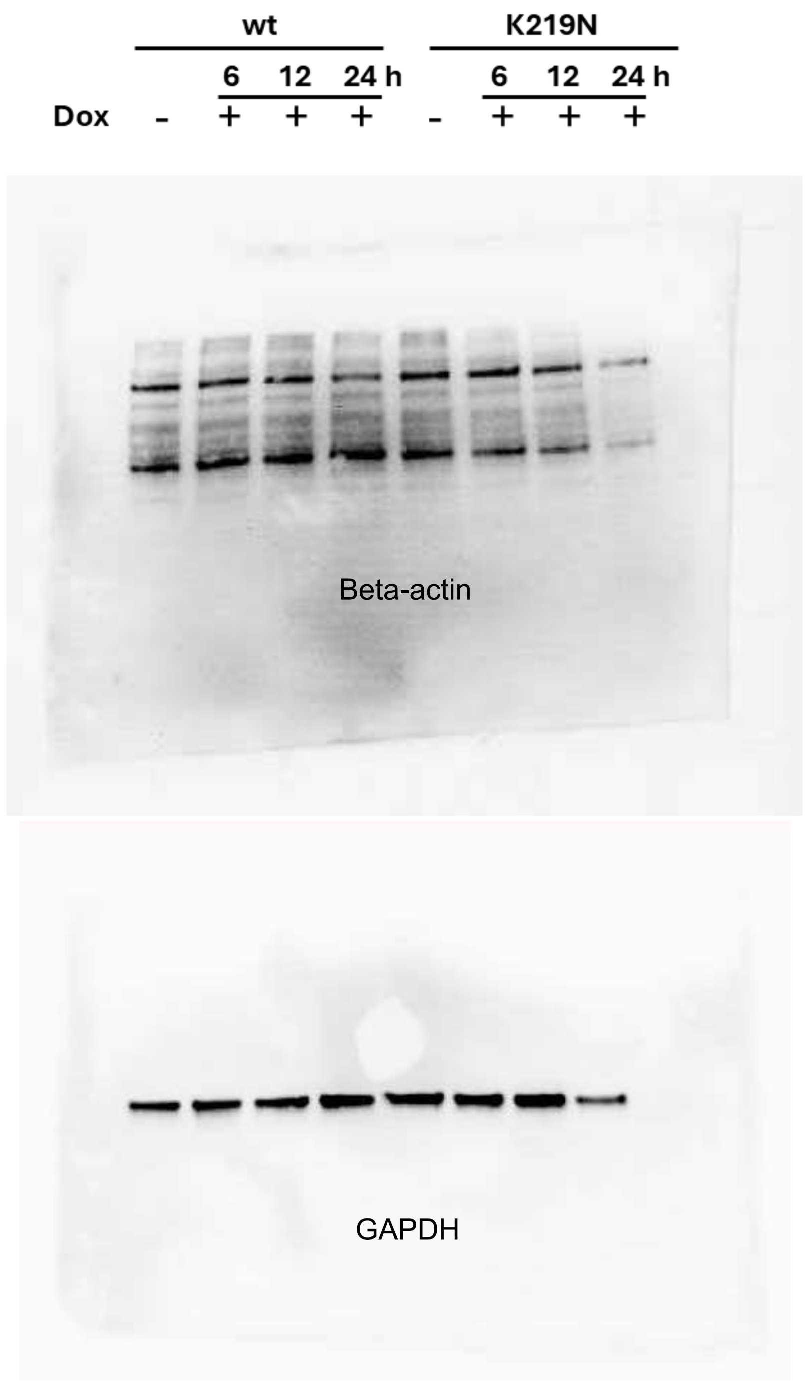
wt
K219N
6 12 24 h
6 12 24 h
Dox - + + + - + + +
Beta-actin
GAPDH

WB

Fig 4B

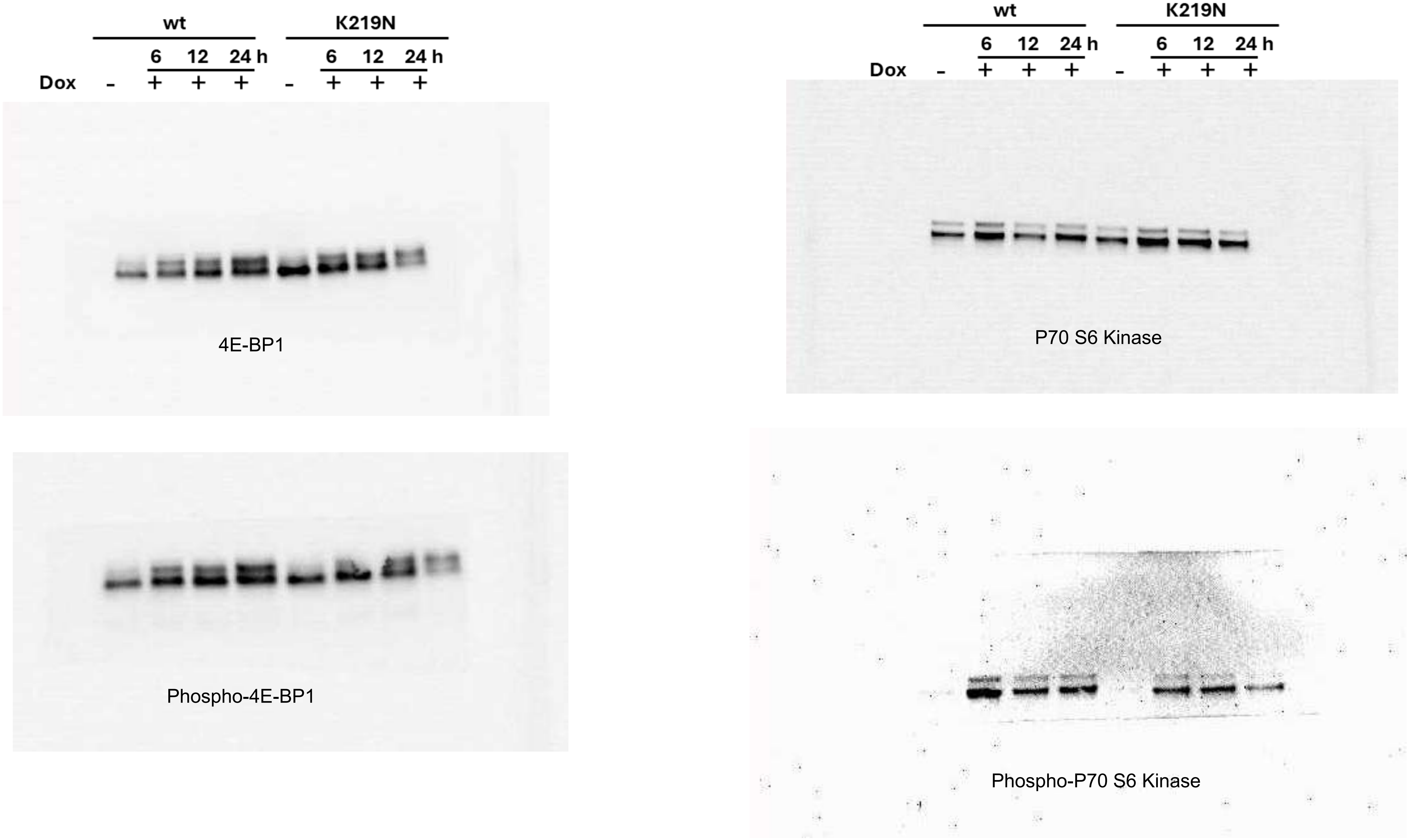
wt
K219N
6 12 24 h
6 12 24 h
Dox - + + + - + + +
4E-BP1
Phospho-4E-BP1
wt
K219N
6 12 24 h
6 12 24 h
Dox - + + + - + + +
P70 S6 Kinase
Phospho-P70 S6 Kinase

WB Fig 4C

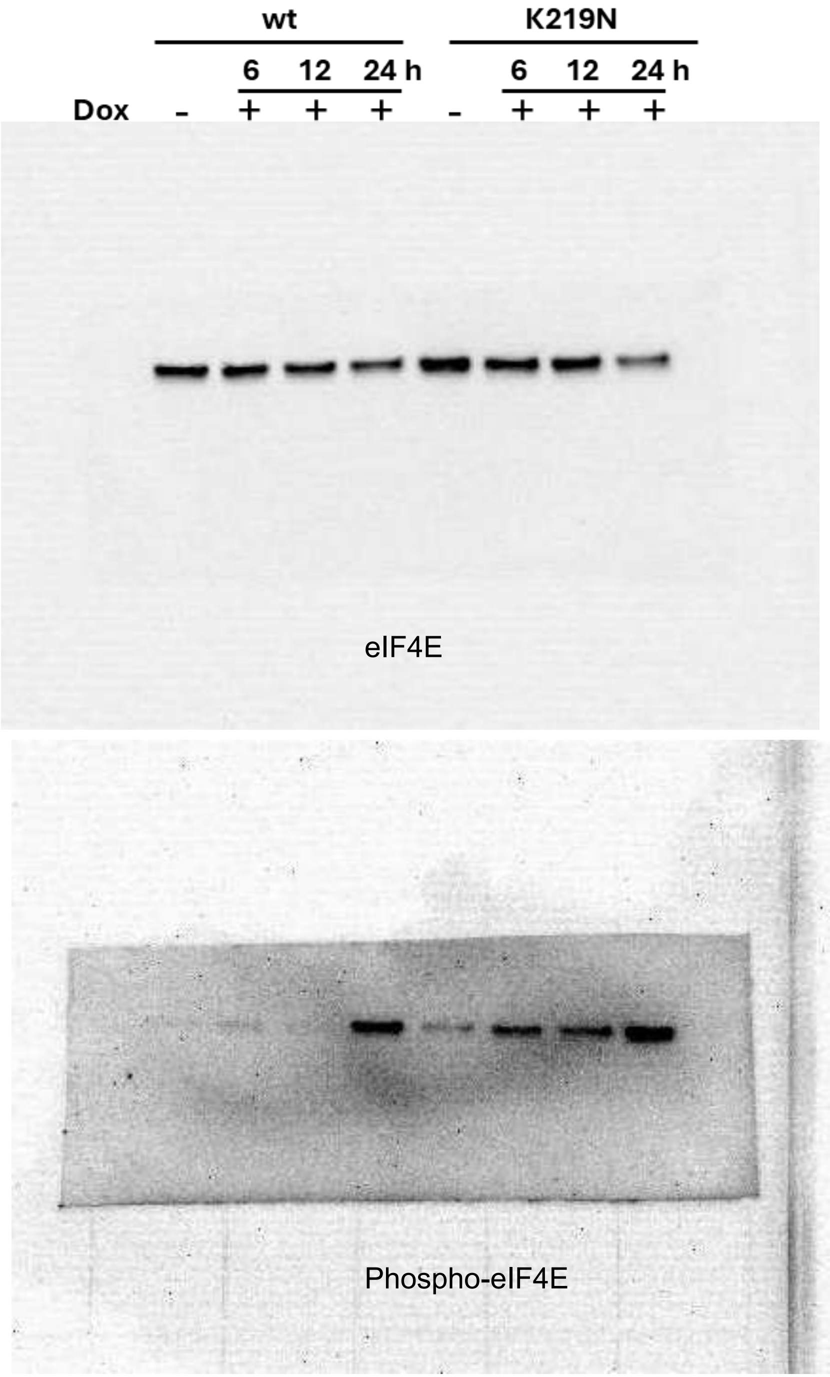

WB

Fig 4D

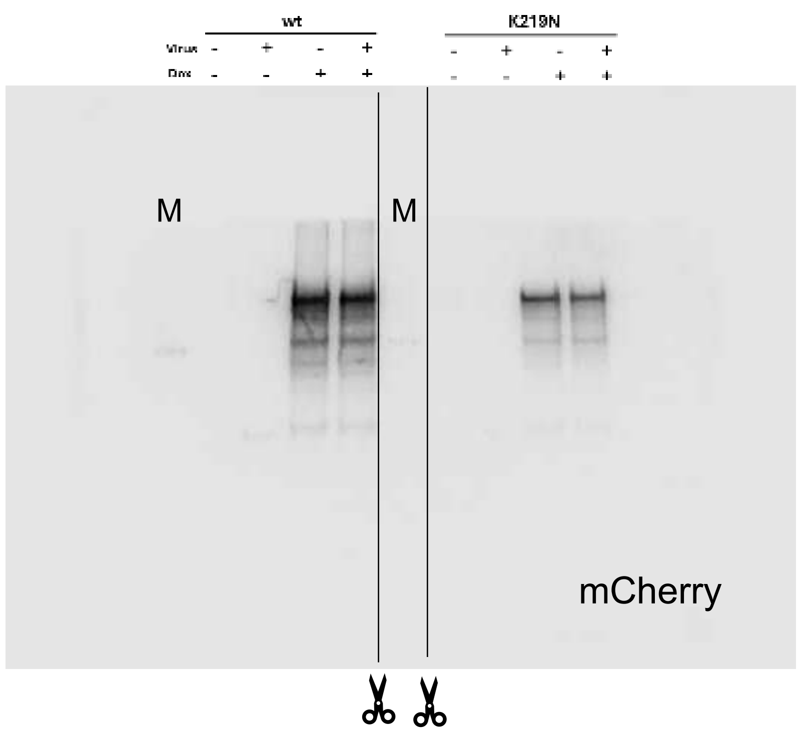

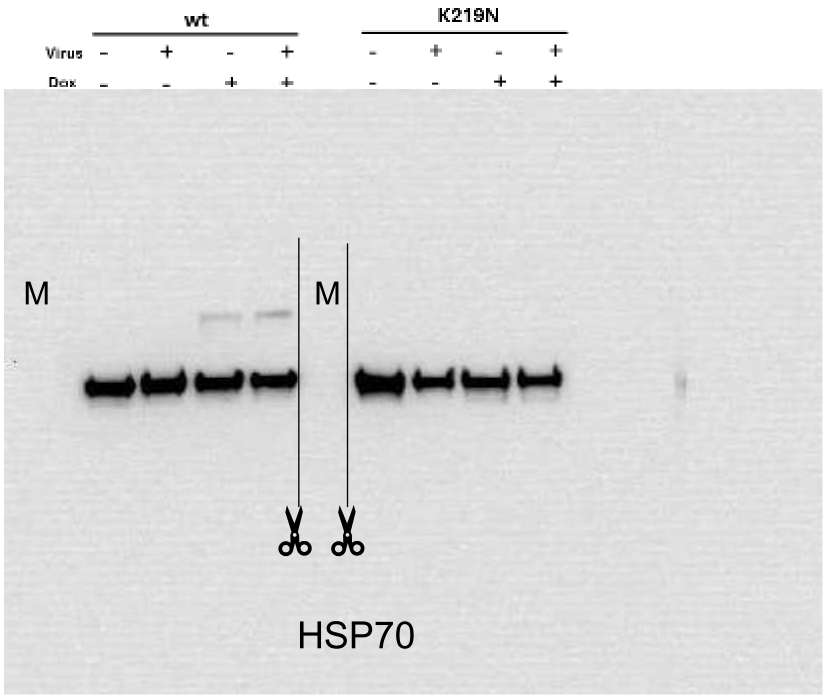

WB Fig S2F
