# Supplementary material for: Type II tRNA cleavage by SLFN14 endoribonuclease variants linked to inherited thrombocytopenia drives global translational repression
Source: PLoS Biol. 2026 May 29;24(5):e3003830. doi: 10.1371/journal.pbio.3003830 (PMC13245857; doi:10.1371/journal.pbio.3003830)
Supplement: Attachment — Submitted filename: Response to Reviewers.docx [file pbio.3003830.s012.docx]

We thank the reviewers for their careful evaluation of our manuscript and for their constructive comments. We have performed additional experiments and substantially revised the manuscript to address all points raised by the reviewers. Major changes include characterization of a catalytically inactive SLFN14 mutant, addressing concerns that N-terminal mCherry tags might affect function, acknowledging the limitations of our HEK293T system, and reconciling the apparent differences with prior publications with respect to rRNA degradation. In addition, we have reorganized the data into four figures, which is the upper limit for a Short Report. We believe this revision has successfully addressed all reviewer comments and meets the editorial request. Below, we respond to each comment in detail.

Reviewer #1: PBIOLOGY-D-26-00347R1
Summary:
The manuscript by Ding et al. proposes that SLFN14 represses global translation through its selective type-II tRNA cleavage activity. Furthermore, the analysis of IT-associated SLFN14 variants suggests altered substrate selectivity, where the variants exhibit a stronger preference for tRNA over rRNA. The authors propose that this shift in substrate preference is directly correlated with enhanced translational arrest, stress signaling, and cell death. To underscore this model, the authors provide a tRNA rescue experiment. Overall, the manuscript is well written. While the results are important to the field, there is key information missing. Please find specific comments below.

1. In general, important insight is missing in the manuscript. For example, how was the amount of doxycycline and corresponding expression level of SLFN14 optimized? Does the expression level mimic the expected level of SLFN14 in megakaryocyte cells or is this an overexpressed condition?  How would SLFN14 overexpression impact the interpretation of subsequent experiments? The authors should comment on these points and provide a Western blot analysis in Figure 1 to demonstrate dox-induced SLFN14 expression at time points used in subsequent experiments.

We thank the reviewer for raising this important point regarding SLFN14 expression levels and their potential impact on the interpretation of our results. We have addressed this concern through clarification of our experimental design and expanding the Discussion.

First, we used both transient transfection and doxycycline-inducible systems to examine SLFN14 function across a range of expression levels. In both cases, SLFN14 expression and its cellular effects were monitored at the single-cell level using flow cytometry. This approach allowed us to analyze cellular phenotypes across a spectrum of SLFN14 expression levels within the same population. As shown in Fig. 1B, cells expressing low levels of WT SLFN14 exhibited little or no inhibition of protein synthesis, whereas even low levels of the K219N variant resulted in marked translational suppression. Thus, single-cell analysis across the population allowed us to assess how cellular phenotypes change as a function of SLFN14 expression level.

Second, we acknowledge that the inducible system may not replicate endogenous SLFN14 expression levels in megakaryocytes. At the same time, we note that SLFN14 is an interferon-stimulated gene and can be induced to higher levels by interferons in many cell types. Therefore, examining SLFN14 function across a range of expression levels provides insight into its activity under both basal and induced states. We have clarified this point in the revised manuscript.

Third, we have included in Figure 4 a Western blot analysis showing doxycycline-induced SLFN14 expression over time (6, 12, and 24 hours).

Finally, we fully acknowledge the limitations of the HEK293T system and have added a paragraph to the Discussion to explicitly address this issue and its implications for physiological relevance. This paragraph is reproduced below:

A limitation of our current study is that all experiments were performed in HEK293T cells and none in megakaryocytes. Studies in HEK293T cells have previously played an important role in defining the molecular function of SLFN14 [5], and this system was chosen here to compare the effects of IT-associated mutations across a wider range of expression levels. However, SLFN14 is constitutively expressed primarily in megakaryocytes and erythroid precursors and is induced in other cell types only under interferon-stimulated conditions. Accordingly, the implications of the SLFN14 functions revealed here for IT pathogenesis should be interpreted with some caution. In a previous study, platelets derived from some IT patients and an immortalized megakaryocyte cell line expressing SLFN14^K219N^ showed increased rRNA degradation [3], although tRNA levels were not examined. By contrast, our studies unexpectedly show that SLFN14^K219N^ is associated with reduced rRNA cleavage and enhanced type II tRNA cleavage in HEK293T cells. It is therefore possible that the extent of rRNA cleavage by SLFN14^K219N^ differs across cell types and experimental conditions. We consider the identification of selective targeting of type II tRNAs by IT-associated SLFN14 variants to be a key contribution of this study and propose that this effect, together with dysregulated rRNA degradation, contributes to pathogenesis. Future studies in megakaryocytes will be important to validate this idea.

2. The authors insert mCherry upstream of SLFN14. In SLFN14's homodimer arrangement, this would result in two ~25 kDa protein moieties proximal to the RNA processing cleft and hotspot mutations tested in this study. Did the authors verify whether mCherry could impose steric hindrance or other structural distortions to the RNA processing cleft? The authors should perform a control experiment where the mCherry is moved to the C-terminus of SLFN14 or the N-terminal mCherry moiety is exchanged for a short epitope tag to ensure the substrate preference switch reported in Figure 3 is independent of their construct design.

We thank the reviewer for raising this point. In our study, mCherry was linked to SLFN14 through a P2A ribosomal skipping sequence, which produces SLFN14 as a separate protein lacking the mCherry moiety. To further address this concern, we tested an additional construct expressing SLFN14 with an N-terminal FLAG tag (FLAG-SLFN14). We now show that FLAG-SLFN14 also inhibited global protein synthesis (in new S1 Fig. D), supporting the conclusion that the functional effects reported here are not an artifact of the mCherry design and are not abolished by N-terminal tagging.

3. The authors draw a direct correlation between enhanced tRNA cleavage and altered ribosome stalling. To make this conclusion, a catalytically inactive mutant (-/+ K219N) would be necessary to support their model that tRNA cleavage itself is necessary.

We appreciate this helpful suggestion. In response, we generated a catalytically inactive E206A mutant in the background of both WT and K219N SLFN14. As shown in the new S1 Fig., the E206A mutation abolishes the ability of both WT and K219N SLFN14 to suppress global protein synthesis and inhibit viral replication. These findings demonstrate that the biological activities described in our study are dependent on SLFN14 RNase activity and provide direct support for the model that RNA cleavage is required for the observed translational and antiviral phenotypes.

4. Figure 1A presents the positions of N-terminal IT-linked SLFN14 residues. Unfortunately, this image is difficult to interpret. The authors should provide an inset so readers can appreciate that V220 is in a distinct local environment from K218, K219, and R223. In fact, this difference in local environment may explain the distinct effect of V220D on global protein synthesis presented in Figure 1.

We thank the reviewer for this insightful suggestion. In response, we have added an inset to Fig. 1A highlighting residues K218, K219, V220, and R223 in the context of the bound RNA. As noted by the reviewer, this view reveals that V220 occupies a distinct local environment compared to K218, K219, and R223, with its side chain oriented away from the RNA-binding interface. This structural distinction is consistent with the differential functional effect of the V220D mutation on global protein synthesis observed in Fig. 1. We have revised the Results section accordingly and now state: “Consistent with this distinction, structural analysis shows that V220 is oriented away from the RNA-binding interface [11], in contrast to K218, K219, and R223 (Fig. 1A).”

5. The authors state "…an AlphaFold3 model of SLFN14 in complex with tRNA-LeuTAA position K219 in close proximity to the bound tRNA (Fig. 3G), providing a structural explanation for the altered substrate specificity observed in the K219N mutant." It is not clear from this statement nor the provided illustration why this provides insight into selectivity. Also, this image is particularly ineffective without the local confidence metrics. Finally, if this image was generated with a type-II tRNA, then the authors should identify the position of the extended stem-loop element.

We agree with the reviewer that the original AlphaFold3 model figure was not effective. We have therefore revised the figure and moved it to Supplemental Fig. S4. As suggested, the revised figure is now colored by local confidence metrics (pLDDT) and includes an enlarged view of the IT-associated residues relative to the bound type II tRNA, with the tRNA variable stem-loop indicated.

We have also moderated our interpretation of the model by removing the statement of the model from the Results section. Instead, we now describe the model cautiously in the Discussion: “How the K219N mutation alters RNA substrate specificity will require further structural and functional studies. Interestingly, an AlphaFold3 [20] model of SLFN14 in complex with tRNA-Leu^TAA^ positions K219 near the tRNA acceptor stem, where cleavage is predicted to occur (S4 Fig).”

6. It is not clear how the authors selected time points for their experiments. For instance, a difference in tRNA cleavage between WT and K219N was observed at 12 hrs. And yet, ribo-seq was performed at 24 hr when there is little difference between WT and K219N. Additionally, the authors later report significant cell death at the 24 hr time point for K219N, further complicating the interpretation of these results.

We welcome the opportunity to clarify our rationale for time point selection. In this study, RNA cleavage was analyzed at 12 h and 26 h after SLFN14 induction, whereas ribo-seq was performed at 24 h after induction. We selected an early time point (12 h) and a later time point (26 h) for RNA cleavage analysis after pilot experiments. We chose only the later time point for ribo-seq because the effects of tRNA depletion were more pronounced for both WT and K219N variant at the later time than at 12 h, and we therefore expected ribosome stalling to be more readily detectable. The small difference between 24 h and 26 h in these experiments reflects experimental logistics and, in our view, does not affect interpretation of the results. Importantly, the ribosome profiling data obtained at 24 h are consistent with the RNA cleavage patterns observed at the closely related 26 h time point. This was particularly evident at serine codons: K219N caused clear stalling at serine codons at 24 h, whereas WT produced little or no stalling at these codons, consistent with the difference in tRNA-Ser depletion between the two samples at 26 h.

We also want to clarify that less than 30% cell death at 24 h did not compromise the ribosome profiling experiment. As described in the method on ribo-seq, cells were plated on poly-D-lysine-coated dishes (0.1 mg/mL) to improve cell adherence, thereby enriching for viable attached cells. In addition, the ribosome profiling library preparation method used here, which we have described recently in several publications, is highly efficient and requires only a small amount of starting material, with approximately 10 ng of ribosome-protected fragments per sample being sufficient for robust library generation. We therefore do not believe that minor cell loss materially affected the quality or interpretability of the ribo-seq data.

7. Northern blot analysis only presents loss of the intact tRNA. SLFN ribonucleases, including SLFN14, have been shown to cleave at a canonical acceptor stem site forming a distinct tRNA product. Did the authors detect this production? Are there differences in the cleavage pattern between WT and K219N? Perhaps the ribo-seq data at 24 hrs reflects differences in the tRNA cleavage pattern?

The point about tRNA cleavage products is an interesting point that we had also considered. However, we did not detect discrete tRNA cleavage products by Northern blot and therefore could not determine whether WT and K219N differ in cleavage pattern. One possible explanation is that the initial cleavage products are rapidly degraded by cellular nucleases and therefore do not accumulate to detectable levels. We observed a similar phenomenon in our previous studies of the tRNA endoribonuclease SAMD9, in which transient cleavage products were rapidly lost, presumably because of processing by additional cellular nucleases.

8. The authors describe tRNA 'degradation'. I caution the authors with this interpretation. Clearance of the tRNA has not been well established in this study and the relevance of the selected SLFN14 expression level may lead to tRNA clearance that is not physiologically relevant.

We appreciate the reviewer’s caution regarding this interpretation. As elaborated in the Discussion, our data establish a strong correlation between the loss of full-length type II tRNAs and the downstream phenotypes of codon-specific ribosome stalling, translational inhibition, and antiviral activity. In addition, the newly added catalytic mutant experiments show that both translational inhibition and antiviral activity depend on SLFN14 RNase activity, further supporting a model in which SLFN14-mediated tRNA cleavage underlies these biological effects. However, we acknowledge that we did not directly detect the end products of tRNA degradation and have therefore revised the manuscript throughout to replace “tRNA degradation” with “tRNA depletion” or “tRNA cleavage,” where appropriate.

We have also addressed the reviewer’s concern regarding SLFN14 expression levels in our response to comment #1.

9. The authors don't appear to provide evidence that their tRNA expression system is producing the corresponding tRNA.

We acknowledge this limitation. The tRNA expression constructs used here are the same as those used successfully in our previous studies of SAMD9, and we have now cited that work in the Methods section. Because of figure limitations for Short Report, and because these data play a relatively minor role in supporting our main conclusions, we have moved the tRNA rescue data from the main figure to Supplemental Fig. S3.

Minor comments:
1. The manuscript could be further strengthened by contextualizing observations. Specifically, the authors should discuss in greater detail how their work aligns with reports on SLFN11 and SLFN12 selectivity.

We agree with the reviewer that placing our findings in the broader context of other SLFN family members strengthens the manuscript. We have a dedicated paragraph in the Discussion placing our SLFN14 findings in a broader framework together with SLFN11 and SLFN12 and advocating a conserved role for type II tRNA targeting across the SLFN family. The relevant paragraph is reproduced below.

The preferential targeting of type II tRNAs by SLFN14 suggests that this is a conserved regulatory strategy within the SLFN family. Although the RNase domains of SLFN11 and SLFN12 display broad RNA substrate specificity in vitro, the full-length proteins selectively target type II tRNAs, particularly tRNA-Leu^TAA^, in cells [21-23]. SLFN14 likewise has broad RNA substrate specificity in vitro and is known to target rRNA in cells, but here we show that it preferentially depletes type II tRNAs in HEK293T cells, indicating that type II tRNAs are a common target for SLFN proteins. SLFN11 is activated in response to DNA damage [22], whereas SLFN12 can be activated by small-molecule “molecular glues” [21]. In contrast, SLFN14 does not appear to require any activation signal beyond transcriptional induction.

2. The authors should explicitly discuss how the use of mCherry, GFP, and housekeeping genes can be used as reporters or to normalize data when SLFN14 drives global translational repression.

We agree with the reviewer that global translational repression by SLFN14 requires careful interpretation of reporter signals and normalization strategies. In this study, we used multiple independent approaches to assess translational inhibition and thereby strengthen the rigor of our conclusions. These included direct measurement of nascent protein synthesis by O-propargyl-puromycin labeling, analysis of fluorescent protein expression across large cell populations, and monitoring of multiple housekeeping proteins by Western blot.

For most assays, we used flow cytometry to simultaneously analyze thousands of cells, allowing us to compare population behaviors while minimizing reliance on normalization. We measured global protein synthesis in large cell populations by O-propargyl-puromycin labeling, thereby reducing dependence on individual protein reporters. mCherry and GFP were used to identify and stratify cells according to SLFN14 expression or infection status in flow cytometry assays. Mean fluorescence intensities measured across large cell populations were then used as a qualitative readout, which reduced reliance on normalization. For Western blot analyses, equal numbers of cells were plated at the start of each experiment, and up to three housekeeping proteins were examined. We recognize that different housekeeping proteins are affected differently under conditions of global translational repression, in part depending on their cellular half-lives. We therefore interpreted these signals cautiously and used them as supportive rather than definitive normalization controls.

We have added the following description to immunoblotting method section of the revised manuscript: “For all conditions within the same experiment, equal numbers of cells were plated at the start of the experiment. Up to three housekeeping proteins (HSP70, β-actin, and GAPDH) were examined, as different proteins may be affected differently by global translational repression mediated by SLFN14, in part depending on their cellular half-lives. Signals from these housekeeping proteins were interpreted as supportive rather than definitive normalization controls.”

We also added the following description to antiviral assay method section of the revised manuscript: “mCherry and GFP were used to identify and stratify cells according to SLFN14 expression or infection status, and mean fluorescence intensities measured across large cell populations were used as a qualitative readout.”

3. Figure 1H should include molecular weights and a time course.

Figure 1H, which shows SLFN14 expression, has been moved to Fig. S2F. We have added molecular weight markers to the figure and described the relevant time point in the legend. In addition, a time course of SLFN14 expression after 6, 12, and 24 h of induction is shown in Fig. 4.

4. The authors fail to acknowledge C-terminal IT-linked mutations in the introduction.

We thank the reviewer for pointing this out. We have revised the Introduction to include the recently reported C-terminal IT-associated variant and now state: “a more recent study identified an IT-associated frameshift variant, T853fs, in the C-terminal helicase domain.”

5. The authors direct the reader to a previously published procedure for generating tRNAs for their viral activity recovery assay. However, this is insufficient. At the very least, the authors should include this citation in the tRNA rescue methods paragraph.

We have added the relevant reference to the tRNA rescue method section, which now begins: “The experiment was performed essentially as described previously in our study of the tRNA-Phe targeting endoribonuclease SAMD9 [25].”

6. The authors should provide the sequence for the mCherry-P2A-SLFN14 construct within the supplemental information.

We have now provided the sequence of mCherry-P2A-SLFN14 plasmid in the supplemental text, as requested.

7. The authors should discuss the limitations of using 293T cells when studying IT-linked SLFN14 disease variants.

We have now added a dedicated paragraph in the Discussion addressing the limitations of using 293T cells, which begins: “A limitation of our current study is that all experiments were performed in HEK293T cells and none in megakaryocytes.”

Reviewer #2: The topic of this study is very important and the experiments are well conducted. Current literature contains conflicting data related to the role of mutant SLFN14 for rRNA and/or tRNA and this effect in relation to the role of mutant SLFN14 for inherited thrombocytopenia.
My major concern is that the experiments in this study have been undertaken in 293T cells using a dox-induced SLFN14 (mutant) expression system.
Patients with SLFN14 mutations only present with thrombocytopenia and no other clinical phenotype has been documented. The most plausible reason for this is that SLFN14 has a very restricted expression pattern (see GTEX data) with exclusive expression in megakaryocytes, platelets and erythroblasts. Probably, this protein has a very important role in the formation of platelets.
To decipher it physiological role, studies in 293T cells and overexpression might result in data that can't be reproduced in these blood cells. Platelets have no nucleus, they contain RNA (including tRNA and rRNA) but this RNA is generated in megakaryocytes during endomitosis. This system is unique and therefore, data should be validated in this physiological cell system as otherwise, it is very difficult to draw conclusions on the role of SLFN14 mutations for inherited thrombocytopenia.
Probably the reason of the conflicting data in literature result form using different cell systems that don't express endogenous SLFN14.

We agree with the reviewer on the importance of physiological context and the relevance of studying SLFN14 in megakaryocyte and platelet lineage cells. We acknowledge that SLFN14 exhibits a highly restricted expression pattern under basal conditions and that megakaryocytes represent the most physiologically relevant system for understanding the mechanisms underlying inherited thrombocytopenia.

In response, we have revised the manuscript to explicitly acknowledge the limitations of our experimental system and to temper the implications of our findings with respect to disease pathogenesis. We have added a dedicated paragraph in the Discussion acknowledging that all experiments were performed in HEK293T cells and that validation in megakaryocyte and platelet lineage models will be important in future studies.

At the same time, we note that SLFN14 is an interferon-inducible gene, and its expression can be induced to higher levels by interferons in cells other than megakaryocytes. Moreover, HEK293T cells have been used in prior studies to define key molecular functions of SLFN14, including its ribosome association and RNase activity, which appear to be conserved across cell types. Our goal in this study was to define the general cellular functions of SLFN14 and to understand how disease-associated mutations alter these functions.

We believe that one of the key contributions of this study is the identification of selective targeting of type II tRNAs by IT-associated SLFN14 variants and its impact on global protein synthesis. While we agree that these findings require validation in more physiological systems before they can be directly linked to IT pathogenesis, they provide important insights that can guide future studies in megakaryocyte and platelet systems.

We believe this contribution fits well within the scope of the PLOS Biology Short Report format, which includes findings that are “fundamentally novel and are more useful when disseminated in their early stages—even if not fully understood—so that the scientific community is free to build upon those intriguing and provocative observations.”

We reproduce the newly added Discussion paragraph below:

A limitation of our current study is that all experiments were performed in HEK293T cells and none in megakaryocytes. Studies in HEK293T cells have previously played an important role in defining the molecular function of SLFN14 [5], and this system was chosen here to compare the effects of IT-associated mutations across a wider range of expression levels. However, SLFN14 is constitutively expressed primarily in megakaryocytes and erythroid precursors and is induced in other cell types only under interferon-stimulated conditions. Accordingly, the implications of the SLFN14 functions revealed here for IT pathogenesis should be interpreted with some caution. In a previous study, platelets derived from some IT patients and an immortalized megakaryocyte cell line expressing SLFN14^K219N^ showed increased rRNA degradation [3], although tRNA levels were not examined. By contrast, our studies unexpectedly show that SLFN14^K219N^ is associated with reduced rRNA cleavage and enhanced type II tRNA cleavage in HEK293T cells. It is therefore possible that the extent of rRNA cleavage by SLFN14^K219N^ differs across cell types and experimental conditions. We consider the identification of selective targeting of type II tRNAs by IT-associated SLFN14 variants to be a key contribution of this study and propose that this effect, together with dysregulated rRNA degradation, contributes to pathogenesis. Future studies in megakaryocytes will be important to validate this idea.

Reviewer #3: SUMMARY:
Class II Schlafen (SLFN) proteins are a family of endoribonucleases known to cleave tRNA or rRNA upon diverse activation signals. SLFN14 has garnered attention for its implication in inherited thrombocytopenia (IT), with pathogenic mutations leading to impaired clotting and platelet differentiation. These mutations have been shown to result in rRNA cleavage and aberrant translation in patient samples and in vitro models, respectively.
This manuscript presents an important discovery: both SLFN14:K219N (an IT related mutation) and wild-type SLFN14 specifically cleave type II RNAs, leading to ribosome stalling at the respective codons. Though this paper answers why SLFN14:K219N impacts translation and expands our knowledge of SLFN14 as an endoribonuclease, I have several questions regarding how the author's findings integrate with the existing SLFN14 literature.

MAJOR POINTS:
1)      The authors report that K219N results in a decreased rRNA degradation. This finding directly contradicts the prevailing hypothesis in the field, namely that K219N leads to increased rRNA decay, thereby enhancing ribosome biogenesis through the mTORC1 pathway. Notably, citation 3 demonstrated that platelet samples from patients harboring the K219N mutation exhibit elevated rRNA degradation when compared to control samples - these data are directly derived from affected patients. Citation 5 further corroborates citation 3, showing increased rRNA decay 24 hours following expression of various SLFN14 constructs. The authors must address how their findings reconcile with these published results, particularly given that the contradicting evidence comes from clinically relevant patient samples. Additionally, in Figure 3D there appears to be rRNA decay (at least of 28S and 18S) after 26 hours of SLFN14:K219N induction, with a major 28S decay product at around 2.5 kb.

We thank the reviewer for highlighting the apparent discrepancy between our results and the prevailing hypothesis in the field. We agree that placing our findings in the context of the existing literature is essential. In response, we have now explicitly addressed the apparent discrepancy in the degree of rRNA degradation between our study and prior publications in the Discussion and propose that differences in cell type, expression level, and experimental conditions likely contribute to this difference. As noted by the reviewer, rRNA degradation remained detectable at later time points in K219N-expressing cells. We therefore now consider the identification of selective targeting of type II tRNAs by IT-associated SLFN14 variants to be the key novel finding of our study and propose that this effect, together with dysregulated rRNA degradation, contributes to pathogenesis. This framework help reconcile our findings with prior observations by suggesting that both rRNA and tRNA targeting can be altered by SLFN14 variants, with their relative contributions to pathogenesis to be determined in future studies in megakaryocytes.

We reproduced relevant section of the discussion below:

In a previous study, platelets derived from some IT patients and an immortalized megakaryocyte cell line expressing SLFN14K219N showed increased rRNA degradation [3], although tRNA levels were not examined. By contrast, our studies unexpectedly show that SLFN14K219N is associated with reduced rRNA cleavage and enhanced type II tRNA cleavage in HEK293T cells. It is therefore possible that the extent of rRNA cleavage by SLFN14K219N differs across cell types and experimental conditions. We consider the identification of selective targeting of type II tRNAs by IT-associated SLFN14 variants to be a key contribution of this study and propose that this effect, together with dysregulated rRNA degradation, contributes to pathogenesis. Future studies in megakaryocytes will be important to validate this idea.

2)      The OPP experiment examining protein expression is a valuable and necessary contribution to understanding the relationship between SLFN14 and protein synthesis. The authors hypothesize that this translational defect results from SLFN14:K219N-mediated cleavage of type II tRNAs, as shown in their ribosome profiling and northern blot data.
In the Discussion, they propose that physiological SLFN14 activity involves cleavage of both rRNA and tRNA, but that the K219N mutation impairs rRNA cleavage, thereby shifting its endonucleolytic activity toward tRNA degradation. They further suggest that this shift leads to aberrant translation. To corroborate this model, the authors could replicate the OPP assay while introducing additional type II tRNA to determine whether this rescues the reduction in total protein production.
Overall, this paper provides important insights into the potential role of SLFN14:K219N in driving pathogenic phenotypes. I appreciate the authors' efforts and believe it is significant that they identify SLFN14 as a type II tRNA endonuclease. This expands our understanding of its impact on translation beyond its previously described role in rRNA cleavage. However, to support the definitive claims made in the Discussion, the manuscript would benefit from the additional experiments outlined above or from tempering some of the conclusions (ex that K219N switches SLFN14 endonucleolytic activity from rRNA to tRNA and that the K219N mutation decreases rRNA cleavage).

We thank the reviewer for this thoughtful comment and for urging caution in our interpretation. In response, we have revised manuscript in many places to temper our conclusions. We do not propose that the K219N mutation switches SLFN14 endonucleolytic activity from rRNA to tRNA. Rather, our data indicate that the K219N variant remains capable of cleaving rRNA, but does so less efficiently than WT under our experimental conditions. We now emphasize that both rRNA and tRNA targeting can be altered by SLFN14 variants, and that their relative contributions to pathogenesis remain to be determined in future studies, particularly in physiologically relevant megakaryocyte systems.
