# Supplementary material for: Type II tRNA cleavage by SLFN14 endoribonuclease variants linked to inherited thrombocytopenia drives global translational repression
Source: PLoS Biol. 2026 May 29;24(5):e3003830. doi: 10.1371/journal.pbio.3003830 (PMC13245857; doi:10.1371/journal.pbio.3003830)
Supplement: Peer review history

# Editor report: Decision Letter 0

**DOI:** 10.1371/journal.pbio.3003830.r001

**Authors:** Richard Hodge

5 Feb 2026

Dear Dr Xiang,

Thank you for submitting your manuscript entitled "Selective targeting of type II tRNAs underlies SLFN14-mediated translational repression and its dysregulation by thrombocytopenia-linked mutations" for consideration as a Research Article by PLOS Biology.

Your manuscript has now been evaluated by the PLOS Biology editorial staff, as well as by an academic editor with relevant expertise, and I am writing to let you know that we would like to send your submission out for external peer review.

IMPORTANT: After discussions with the academic editor, we would like to consider your manuscript as a 'Short Report' at the journal (https://journals.plos.org/plosbiology/s/what-we-publish#loc-short-reports). Upon resubmission (see guidelines below), I would be grateful if you could please tick 'Short Report' as the article type in the dropdown menu of the online submission form.

Before we can send your manuscript to reviewers, we need you to complete your submission by providing the metadata that is required for full assessment. To this end, please login to Editorial Manager where you will find the paper in the 'Submissions Needing Revisions' folder on your homepage. Please click 'Revise Submission' from the Action Links and complete all additional questions in the submission questionnaire.

Once your full submission is complete, your paper will undergo a series of checks in preparation for peer review. After your manuscript has passed the checks it will be sent out for review. To provide the metadata for your submission, please Login to Editorial Manager (https://www.editorialmanager.com/pbiology) within two working days, i.e. by Feb 07 2026 11:59PM.

If your manuscript has been previously peer-reviewed at another journal, PLOS Biology is willing to work with those reviews in order to avoid re-starting the process. Submission of the previous reviews is entirely optional and our ability to use them effectively will depend on the willingness of the previous journal to confirm the content of the reports and share the reviewer identities. Please note that we reserve the right to invite additional reviewers if we consider that additional/independent reviewers are needed, although we aim to avoid this as far as possible. In our experience, working with previous reviews does save time.

If you would like us to consider previous reviewer reports, please edit your cover letter to let us know and include the name of the journal where the work was previously considered and the manuscript ID it was given. In addition, please upload a response to the reviews as a 'Prior Peer Review' file type, which should include the reports in full and a point-by-point reply detailing how you have or plan to address the reviewers' concerns.

During the process of completing your manuscript submission, you will be invited to opt-in to posting your pre-review manuscript as a bioRxiv preprint. Visit http://journals.plos.org/plosbiology/s/preprints for full details. If you consent to posting your current manuscript as a preprint, please upload a single Preprint PDF.

Feel free to email us at plosbiology@plos.org if you have any queries relating to your submission.

Kind regards,

Richard

Richard Hodge, PhD

Senior Editor, PLOS Biology

rhodge@plos.org

PLOS

Empowering researchers to transform science

Carlyle House, Carlyle Road, Cambridge, CB4 3DN, United Kingdom

California (U.S.) corporation #C2354500, based in San Francisco

# Peer review: Decision Letter 1

**DOI:** 10.1371/journal.pbio.3003830.r002

**Authors:** Richard Hodge

15 Mar 2026

Dear Dr Xiang,

Thank you for your patience while your manuscript "Selective targeting of type II tRNAs underlies SLFN14-mediated translational repression and its dysregulation by thrombocytopenia-linked mutations" was peer-reviewed at PLOS Biology. Please accept my sincere apologies for the delays that you have experienced during the peer review process. Your manuscript has now been evaluated by the PLOS Biology editors, an Academic Editor with relevant expertise, and by three independent reviewers.

In light of the reviews, which you will find at the end of this email, we would like to invite you to revise the work to thoroughly address the reviewers' reports.

As you will see below, the reviewers agree that the findings are potentially interesting for the field but they each raise several concerns. Reviewer #1 notes that important control experiments and reporting details are missing, including data using a catalytically inactive SLFN14 mutant to fully support the model that tRNA cleavage is necessary for altered ribosome stalling, as well as controlling for potential artefacts induced by addition of the mCherry tag. Reviewer #2 is more negative and raises concerns with the overall physiological relevance of the work given that a HEK293T model system is used. In a revised version, we ask that this limitation is fully acknowledged and discussed in detail. Reviewer #3 notes that several of the findings contradict previous work, such as the SLFN14-K219N mutant leading to increased rRNA decay, so these discrepancies should be acknowledged and prior work sufficiently contextualized.

In addition, we are considering your manuscript as a Short Report at the journal (https://journals.plos.org/plosbiology/s/what-we-publish#loc-short-reports). Our Short Report's have a maximum of 4 main figures, so we ask that you please reduce the number of main figures at this stage by either combining main figures or moving some figures to the supplementary.

Given the extent of revision needed, we cannot make a decision about publication until we have seen the revised manuscript and your response to the reviewers' comments. Your revised manuscript is likely to be sent for further evaluation by all or a subset of the reviewers.

In addition to these revisions, you will need to complete some formatting changes, which you will receive in a follow up email. A member of our team will be in touch with a set of requests shortly.

We expect to receive your revised manuscript within 3 months. Please email us (plosbiology@plos.org) if you have any questions or concerns, or would like to request an extension.

At this stage, your manuscript remains formally under active consideration at our journal; please notify us by email if you do not intend to submit a revision so that we may withdraw it.

**IMPORTANT - SUBMITTING YOUR REVISION**

Your revisions should address the specific points made by each reviewer. Please submit the following files along with your revised manuscript:

1. A 'Response to Reviewers' file - this should detail your responses to the editorial requests, present a point-by-point response to all of the reviewers' comments, and indicate the changes made to the manuscript.

*NOTE: In your point-by-point response to the reviewers, please provide the full context of each review. Do not selectively quote paragraphs or sentences to reply to. The entire set of reviewer comments should be present in full and each specific point should be responded to individually, point by point.

You should also cite any additional relevant literature that has been published since the original submission and mention any additional citations in your response.

2. In addition to a clean copy of the manuscript, please also upload a 'track-changes' version of your manuscript that specifies the edits made. This should be uploaded as a "Revised Article with Changes Highlighted" file type.

*Re-submission Checklist*

When you are ready to resubmit your revised manuscript, please refer to this re-submission checklist: https://plos.io/Biology_Checklist

To submit a revised version of your manuscript, please go to https://www.editorialmanager.com/pbiology/ and log in as an Author. Click the link labelled 'Submissions Needing Revision' where you will find your submission record.

Please make sure to read the following important policies and guidelines while preparing your revision:

*Published Peer Review*

Please note while forming your response, if your article is accepted, you may have the opportunity to make the peer review history publicly available. The record will include editor decision letters (with reviews) and your responses to reviewer comments. If eligible, we will contact you to opt in or out. Please see here for more details:

https://blogs.plos.org/plos/2019/05/plos-journals-now-open-for-published-peer-review/

*PLOS Data Policy*

Please note that as a condition of publication PLOS' data policy (http://journals.plos.org/plosbiology/s/data-availability) requires that you make available all data used to draw the conclusions arrived at in your manuscript. If you have not already done so, you must include any data used in your manuscript either in appropriate repositories, within the body of the manuscript, or as supporting information (N.B. this includes any numerical values that were used to generate graphs, histograms etc.). For an example see here: http://www.plosbiology.org/article/info%3Adoi%2F10.1371%2Fjournal.pbio.1001908#s5

*Blot and Gel Data Policy*

We require the original, uncropped and minimally adjusted images supporting all blot and gel results reported in an article's figures or Supporting Information files. We will require these files before a manuscript can be accepted so please prepare them now, if you have not already uploaded them. Please carefully read our guidelines for how to prepare and upload this data: https://journals.plos.org/plosbiology/s/figures#loc-blot-and-gel-reporting-requirements

*Protocols deposition*

To enhance the reproducibility of your results, we recommend that if applicable you deposit your laboratory protocols in protocols.io, where a protocol can be assigned its own identifier (DOI) such that it can be cited independently in the future. Additionally, PLOS ONE offers an option for publishing peer-reviewed Lab Protocol articles, which describe protocols hosted on protocols.io. Read more information on sharing protocols at https://plos.org/protocols?utm_medium=editorial-email&utm_source=authorletters&utm_campaign=protocols

Thank you again for your submission to our journal. We hope that our editorial process has been constructive thus far, and we welcome your feedback at any time. Please don't hesitate to contact us if you have any questions or comments.

Best regards,

Richard

Richard Hodge, PhD

Senior Editor, PLOS Biology

rhodge@plos.org

------------------------------------

REVIEWS:

Reviewer #1: PBIOLOGY-D-26-00347R1

Summary:

The manuscript by Ding et al. proposes that SLFN14 represses global translation through its selective type-II tRNA cleavage activity. Furthermore, the analysis of IT-associated SLFN14 variants suggests altered substrate selectivity, where the variants exhibit a stronger preference for tRNA over rRNA. The authors propose that this shift in substrate preference is directly correlated with enhanced translational arrest, stress signaling, and cell death. To underscore this model, the authors provide a tRNA rescue experiment. Overall, the manuscript is well written. While the results are important to the field, there is key information missing. Please find specific comments below.

1. In general, important insight is missing in the manuscript. For example, how was the amount of doxycycline and corresponding expression level of SLFN14 optimized? Does the expression level mimic the expected level of SLFN14 in megakaryocyte cells or is this an overexpressed condition? How would SLFN14 overexpression impact the interpretation of subsequent experiments? The authors should comment on these points and provide a Western blot analysis in Figure 1 to demonstrate dox-induced SLFN14 expression at time points used in subsequent experiments.

2. The authors insert mCherry upstream of SLFN14. In SLFN14's homodimer arrangement, this would result in two ~25 kDa protein moieties proximal to the RNA processing cleft and hotspot mutations tested in this study. Did the authors verify whether mCherry could impose steric hindrance or other structural distortions to the RNA processing cleft? The authors should perform a control experiment where the mCherry is moved to the C-terminus of SLFN14 or the N-terminal mCherry moiety is exchanged for a short epitope tag to ensure the substrate preference switch reported in Figure 3 is independent of their construct design.

3. The authors draw a direct correlation between enhanced tRNA cleavage and altered ribosome stalling. To make this conclusion, a catalytically inactive mutant (-/+ K219N) would be necessary to support their model that tRNA cleavage itself is necessary.

4. Figure 1A presents the positions of N-terminal IT-linked SLFN14 residues. Unfortunately, this image is difficult to interpret. The authors should provide an inset so readers can appreciate that V220 is in a distinct local environment from K218, K219, and R223. In fact, this difference in local environment may explain the distinct effect of V220D on global protein synthesis presented in Figure 1.

5. The authors state "…an AlphaFold3 model of SLFN14 in complex with tRNA-LeuTAA position K219 in close proximity to the bound tRNA (Fig. 3G), providing a structural explanation for the altered substrate specificity observed in the K219N mutant." It is not clear from this statement nor the provided illustration why this provides insight into selectivity. Also, this image is particularly ineffective without the local confidence metrics. Finally, if this image was generated with a type-II tRNA, then the authors should identify the position of the extended stem-loop element.

6. It is not clear how the authors selected time points for their experiments. For instance, a difference in tRNA cleavage between WT and K219N was observed at 12 hrs. And yet, ribo-seq was performed at 24 hr when there is little difference between WT and K219N. Additionally, the authors later report significant cell death at the 24 hr time point for K219N, further complicating the interpretation of these results.

7. Northern blot analysis only presents loss of the intact tRNA. SLFN ribonucleases, including SLFN14, have been shown to cleave at a canonical acceptor stem site forming a distinct tRNA product. Did the authors detect this production? Are there differences in the cleavage pattern between WT and K219N? Perhaps the ribo-seq data at 24 hrs reflects differences in the tRNA cleavage pattern?

8. The authors describe tRNA 'degradation'. I caution the authors with this interpretation. Clearance of the tRNA has not been well established in this study and the relevance of the selected SLFN14 expression level may lead to tRNA clearance that is not physiologically relevant.

9. The authors don't appear to provide evidence that their tRNA expression system is producing the corresponding tRNA.

Minor comments:

1. The manuscript could be further strengthened by contextualizing observations. Specifically, the authors should discuss in greater detail how their work aligns with reports on SLFN11 and SLFN12 selectivity.

2. The authors should explicitly discuss how the use of mCherry, GFP, and housekeeping genes can be used as reporters or to normalize data when SLFN14 drives global translational repression.

3. Figure 1H should include molecular weights and a time course.

4. The authors fail to acknowledge C-terminal IT-linked mutations in the introduction.

5. The authors direct the reader to a previously published procedure for generating tRNAs for their viral activity recovery assay. However, this is insufficient. At the very least, the authors should include this citation in the tRNA rescue methods paragraph.

6. The authors should provide the sequence for the mCherry-P2A-SLFN14 construct within the supplemental information.

7. The authors should discuss the limitations of using 293T cells when studying IT-linked SLFN14 disease variants.

Reviewer #2: The topic of this study is very important and the experiments are well conducted. Current literature contains conflicting data related to the role of mutant SLFN14 for rRNA and/or tRNA and this effect in relation to the role of mutant SLFN14 for inherited thrombocytopenia.

My major concern is that the experiments in this study have been undertaken in 293T cells using a dox-induced SLFN14 (mutant) expression system.

Patients with SLFN14 mutations only present with thrombocytopenia and no other clinical phenotype has been documented. The most plausible reason for this is that SLFN14 has a very restricted expression pattern (see GTEX data) with exclusive expression in megakaryocytes, platelets and erythroblasts. Probably, this protein has a very important role in the formation of platelets.

To decipher it physiological role, studies in 293T cells and overexpression might result in data that can't be reproduced in these blood cells. Platelets have no nucleus, they contain RNA (including tRNA and rRNA) but this RNA is generated in megakaryocytes during endomitosis. This system is unique and therefore, data should be validated in this physiological cell system as otherwise, it is very difficult to draw conclusions on the role of SLFN14 mutations for inherited thrombocytopenia.

Probably the reason of the conflicting data in literature result form using different cell systems that don't express endogenous SLFN14.

Reviewer #3: SUMMARY:

Class II Schlafen (SLFN) proteins are a family of endoribonucleases known to cleave tRNA or rRNA upon diverse activation signals. SLFN14 has garnered attention for its implication in inherited thrombocytopenia (IT), with pathogenic mutations leading to impaired clotting and platelet differentiation. These mutations have been shown to result in rRNA cleavage and aberrant translation in patient samples and in vitro models, respectively.

This manuscript presents an important discovery: both SLFN14:K219N (an IT related mutation) and wild-type SLFN14 specifically cleave type II RNAs, leading to ribosome stalling at the respective codons. Though this paper answers why SLFN14:K219N impacts translation and expands our knowledge of SLFN14 as an endoribonuclease, I have several questions regarding how the author's findings integrate with the existing SLFN14 literature.

MAJOR POINTS:

1)The authors report that K219N results in a decreased rRNA degradation. This finding directly contradicts the prevailing hypothesis in the field, namely that K219N leads to increased rRNA decay, thereby enhancing ribosome biogenesis through the mTORC1 pathway. Notably, citation 3 demonstrated that platelet samples from patients harboring the K219N mutation exhibit elevated rRNA degradation when compared to control samples - these data are directly derived from affected patients. Citation 5 further corroborates citation 3, showing increased rRNA decay 24 hours following expression of various SLFN14 constructs. The authors must address how their findings reconcile with these published results, particularly given that the contradicting evidence comes from clinically relevant patient samples. Additionally, in Figure 3D there appears to be rRNA decay (at least of 28S and 18S) after 26 hours of SLFN14:K219N induction, with a major 28S decay product at around 2.5 kb.

2)The OPP experiment examining protein expression is a valuable and necessary contribution to understanding the relationship between SLFN14 and protein synthesis. The authors hypothesize that this translational defect results from SLFN14:K219N-mediated cleavage of type II tRNAs, as shown in their ribosome profiling and northern blot data.

In the Discussion, they propose that physiological SLFN14 activity involves cleavage of both rRNA and tRNA, but that the K219N mutation impairs rRNA cleavage, thereby shifting its endonucleolytic activity toward tRNA degradation. They further suggest that this shift leads to aberrant translation. To corroborate this model, the authors could replicate the OPP assay while introducing additional type II tRNA to determine whether this rescues the reduction in total protein production.

Overall, this paper provides important insights into the potential role of SLFN14:K219N in driving pathogenic phenotypes. I appreciate the authors' efforts and believe it is significant that they identify SLFN14 as a type II tRNA endonuclease. This expands our understanding of its impact on translation beyond its previously described role in rRNA cleavage. However, to support the definitive claims made in the Discussion, the manuscript would benefit from the additional experiments outlined above or from tempering some of the conclusions (ex that K219N switches SLFN14 endonucleolytic activity from rRNA to tRNA and that the K219N mutation decreases rRNA cleavage).

# Author comment: Author response to Decision Letter 2

**DOI:** 10.1371/journal.pbio.3003830.r003

2 Apr 2026

Attachment Submitted filename: Response to Reviewers.docx

# Peer review: Decision Letter 2

**DOI:** 10.1371/journal.pbio.3003830.r004

**Authors:** Richard Hodge

8 May 2026

Dear Dr Xiang,

Thank you for your patience while we considered your revised manuscript "Selective targeting of type II tRNAs underlies SLFN14-mediated translational repression and its dysregulation by thrombocytopenia-linked mutations" for publication as a Short Report at PLOS Biology. Please accept my apologies for the delays that you have experienced during this round of the peer review process. This revised version of your manuscript has been evaluated by the PLOS Biology editors, the Academic Editor and two of the the original reviewers.

Based on the reviews, I am pleased to say that we are likely to accept this manuscript for publication, provided you satisfactorily address the following data and other policy-related requests that I have provided below (A-I):

(A) We routinely suggest changes to titles to ensure maximum accessibility for a broad, non-specialist readership. In this case, we would suggest a minor edit to the title as follows, given the comments from Reviewer #3. Please ensure you change both the manuscript file and the online submission system, as they need to match for final acceptance:

“Type II tRNA cleavage by SLFN14 endoribonuclease variants linked to inherited thrombocytopenia drives global translational repression”

(B) In the Financial Disclosure statement in the online submission form, please provide additional details about the funding received to conduct the study (i.e. any grant/funding numbers).

(C) Thank you for already providing the underlying data for the figures in the Supporting Information file ‘data for Graph’. I would be grateful if this could be renamed ‘S1_Data’ so it is consistent with journal style.

(D) In addition, for figures containing FACS data (Figure 1B-C, 1F, S1B-D, S2A, S2C, S3A-B) we ask that you provide FCS files and a picture showing the successive plots and gates that were applied to the FCS files to generate the figure. Please deposit this data in the FlowRepository (https://flowrepository.org/) or the Zenodo repository (https://zenodo.org/) and provide the accession number/URL of the deposition in the Data Availability Statement in the online submission form.

(E) Thank you already providing the uncropped images for the Western blots in the ‘S1_raw_images’ file. This looks good, but I noted that the image for Figure S2F is mislabeled?

(F) Please also ensure that each of the relevant figure legends in your manuscript include information on *WHERE THE UNDERLYING DATA CAN BE FOUND*, and ensure your supplemental data file/s has a legend.

(G) Per journal policy, if you have generated any custom code during the course of this investigation, please make it available without restrictions. Please ensure that the code is sufficiently well documented and reusable, and that your Data Statement in the Editorial Manager submission system accurately describes where your code can be found. More information on our Code Policy, what and how to share can be found here: https://journals.plos.org/plosbiology/s/code-availability

Please note that we cannot accept sole deposition of code in GitHub, as this could be changed after publication. However, you can archive this version of your publicly available GitHub code to Zenodo. Once you do this, it will generate a DOI number, which you will need to provide in the Data Accessibility Statement (you are welcome to also provide the GitHub access information). See the process for doing this here: https://docs.github.com/en/repositories/archiving-a-github-repository/referencing-and-citing-content

(H) Please ensure that you are using best practice for statistical reporting and data presentation, for both the main and supplementary figures. These are our guidelines https://journals.plos.org/plosbiology/s/best-practices-in-research-reporting#loc-statistical-reporting and a useful resource on data presentation https://journals.plos.org/plosbiology/article?id=10.1371/journal.pbio.1002128

- If you are reporting experiments where n ≤ 5, please plot each individual data point.

(I) Please note that per journal policy, the model system/species studied should be clearly stated in the abstract of your manuscript.

------------------------------------------------------------------------

As you address these items, please take this last chance to review your reference list to ensure that it is complete and correct. If you have cited papers that have been retracted, please include the rationale for doing so in the manuscript text, or remove these references and replace them with relevant current references. Any changes to the reference list should be mentioned in the cover letter that accompanies your revised manuscript.

In addition to these revisions, you may need to complete some formatting changes, which you will receive in a follow up email. A member of our team will be in touch with a set of requests shortly. If you do not receive a separate email within a few days, please assume that checks have been completed, and no additional changes are required.

We expect to receive your revised manuscript within two weeks.

To submit your revision, please go to https://www.editorialmanager.com/pbiology/ and log in as an Author. Click the link labelled 'Submissions Needing Revision' to find your submission record. Your revised submission must include the following:

- a cover letter that should detail your responses to any editorial requests, if applicable, and whether changes have been made to the reference list

- a Response to Reviewers file that provides a detailed response to the reviewers' comments (if applicable, if not applicable please do not delete your existing 'Response to Reviewers' file.)

- a track-changes file indicating any changes that you have made to the manuscript.

NOTE: If Supporting Information files are included with your article, note that these are not copyedited and will be published as they are submitted. Please ensure that these files are legible and of high quality (at least 300 dpi) in an easily accessible file format. For this reason, please be aware that any references listed in an SI file will not be indexed. For more information, see our Supporting Information guidelines:

https://journals.plos.org/plosbiology/s/supporting-information

*Published Peer Review History*

Please note that you may have the opportunity to make the peer review history publicly available. The record will include editor decision letters (with reviews) and your responses to reviewer comments. If eligible, we will contact you to opt in or out. Please see here for more details:

https://plos.org/published-peer-review-history/

*Press*

Should you, your institution's press office or the journal office choose to press release your paper, please ensure you have opted out of Early Article Posting on the submission form. We ask that you notify us as soon as possible if you or your institution is planning to press release the article.

*Protocols deposition*

To enhance the reproducibility of your results, we recommend that if applicable you deposit your laboratory protocols in protocols.io, where a protocol can be assigned its own identifier (DOI) such that it can be cited independently in the future. Additionally, PLOS ONE offers an option for publishing peer-reviewed Lab Protocol articles, which describe protocols hosted on protocols.io. Read more information on sharing protocols at https://plos.org/protocols?utm_medium=editorial-email&utm_source=authorletters&utm_campaign=protocols

Please do not hesitate to contact me should you have any questions.

Best regards,

Richard

Richard Hodge, PhD

Senior Editor, PLOS Biology

rhodge@plos.org

------------------------------------------------------------------------

Reviewer remarks:

Reviewer #1: PBIOLOGY-D-26-00347

The authors have revised the manuscript by Ding et al. to address the concerns and suggestions raised during the review. I believe the additional data and updated discussion have greatly improved this study. I support publication of this work.

Reviewer #3: The authors have adequately addressed my concerns. However, I recommend revising the title to better reflect the limitations of the study.

# Editor report: Decision Letter 3

**DOI:** 10.1371/journal.pbio.3003830.r005

**Authors:** Richard Hodge

15 May 2026

Dear Dr Xiang,

Thank you for the submission of your revised Short Report "Type II tRNA cleavage by SLFN14 endoribonuclease variants linked to inherited thrombocytopenia drives global translational repression" for publication in PLOS Biology, and thank you for addressing our editorial requests in this revision. On behalf of my colleague, Richard Hodge (who again, is out of the office at the moment) and the Academic Editor, Wendy V Gilbert, I am pleased to say that we can in principle accept your manuscript for publication, provided you address any remaining formatting and reporting issues. These will be detailed in an email you should receive within 2-3 business days from our colleagues in the journal operations team; no action is required from you until then. Please note that we will not be able to formally accept your manuscript and schedule it for publication until you have completed any requested changes.

**IMPORTANT: As discussed over email, I have updated your Financial Disclosures statement, in our online system, to include the project ID that you provided me. Please take a moment to double check that everything looks good after this change.

Please also take a minute to log into Editorial Manager at http://www.editorialmanager.com/pbiology/, click the "Update My Information" link at the top of the page, and update your user information to ensure an efficient production process.

PRESS

We frequently collaborate with press offices. If your institution or institutions have a press office, please notify them about your upcoming paper at this point, to enable them to help maximise its impact. If the press office is planning to promote your findings, we would be grateful if they could coordinate with biologypress@plos.org. If you have previously opted in to the early version process, we ask that you notify us immediately of any press plans so that we may opt out on your behalf.

We also ask that you take this opportunity to read our Embargo Policy regarding the discussion, promotion and media coverage of work that is yet to be published by PLOS. As your manuscript is not yet published, it is bound by the conditions of our Embargo Policy. Please be aware that this policy is in place both to ensure that any press coverage of your article is fully substantiated and to provide a direct link between such coverage and the published work. For full details of our Embargo Policy, please visit http://www.plos.org/about/media-inquiries/embargo-policy/.

Thank you again for choosing PLOS Biology for publication and supporting Open Access publishing. We look forward to publishing your study.

Sincerely,

Luke Smith, PhD

Senior Editor

PLOS Biology

lsmith@plos.org

---on behalf of---

Richard Hodge, PhD

Senior Editor

PLOS Biology

rhodge@plos.org
